# Angioedemas associated with renin-angiotensin system blocking drugs: Comparative analysis of spontaneous adverse drug reaction reports

**DOI:** 10.1371/journal.pone.0230632

**Published:** 2020-03-26

**Authors:** Diana Dubrall, Matthias Schmid, Julia Carolin Stingl, Bernhardt Sachs

**Affiliations:** 1 Institute for Medical Biometry, Informatics and Epidemiology, (IMBIE), University Hospital of Bonn, North Rhine-Westphalia, Germany; 2 Federal Institute for Drugs and Medical Devices (BfArM), Research Division, North Rhine-Westphalia, Germany; 3 Institute of Clinical Pharmacology, University Hospital of the RWTH Aachen, North Rhine-Westphalia, Germany; 4 Department for Dermatology and Allergy, University Hospital of the RWTH Aachen, North Rhine-Westphalia, Germany; Ehime University Graduate School of Medicine, JAPAN

## Abstract

**Introduction:**

Angioedema is a subcutaneous swelling typically affecting the face, larynx or pharynx. It is a known adverse drug reaction (ADR) of ACE inhibitors (ACEi), angiotensin-II-receptor blockers (ARBs) and aliskiren (renin inhibitor). Several studies have reported pathophysiological mechanisms and risk factors of ACEi-associated angioedemas, whereas little is known for ARBs and aliskiren. The aim of the study was to analyze comparatively ACEi versus ARBs and aliskiren angioedema reports contained in the European ADR database EudraVigilance with regard to reported risk factors and clinical phenotypes.

**Methods:**

All spontaneous angioedema reports received between 01/2010-06/2017 reporting either an ACEi, ARB, or aliskiren as "suspected/interacting" drug were identified using the Standardized MedDRA Query "angioedema (narrow)". In order to perform a comparative analysis, odds ratios (ORs) were calculated for angioedema reports of ACEi (n = 3.194) versus ARBs (n = 687) and aliskiren (n = 162).

**Results:**

More patients with a history of allergy were included in angioedema reports of ARBs (6.8%) and aliskiren (13.6%) versus ACEi (4.3%). "Urticaria" as an ADR was reported more frequently in angioedema reports of ARBs (18.5%) and aliskiren (9.0%) versus ACEi (5.0%). ACEi-associated angioedemas were more often designated as "life-threatening" compared to ARBs (OR 2.2 [1.6–2.9]) and aliskiren-associated angioedemas (OR 14.2 (3.5–57.4). Concomitant therapy with mTOR inhibitors (OR 4.3 [1.0–17.9]) and fibrinolytics (OR 7.8 [1.1–57.2]) was reported more often in ACEi versus ARBs angioedema reports.

**Conclusion:**

The reported clinical phenotypes differed between ACEi versus ARBs and aliskiren angioedema reports. Differences between the patient populations as observed in our study or differences with regard to underlying pathomechanisms could account for this finding. Due to the methodological limitations of spontaneous reporting systems, we cannot draw a firm conclusion in this regard. Hence, further research is necessary to confirm our observation and elucidate the underyling causes.

## Introduction

Angioedema is a deep dermal, subcutaneous swelling that typically affects the face, lips, tongue, larynx or pharynx [1, 2]. It may be life-threatening [1, 3, 4], especially when the airways are involved. Angioedema is a known adverse drug reaction (ADR) for drugs acting on the renin-angiotensin system (RAS) with varying incidences for the individual drug classes.

For instance, about 0.1 to 0.7% of patients treated with angiotensin-converting enzyme inhibitors (ACEi) develop angioedema [3, 5]. In two-thirds of the patients, ACEi angioedemas occurred within the first three months of treatment [6–8]. A multicenter study in the USA [9] estimated that 30% of all emergency department visits due to angioedema are ACEi-associated.

The assumed underlying pathomechanism of ACEi-associated angioedema is the accumulation of bradykinin through inhibition of ACE (angiotensin converting enzyme). ACE is the mainly responsible enzyme for the degradation of bradykinin [10]. If other bradykinin degrading enzymes cannot compensate for this inhibition due to functional relevant genetic variants or environmental factors [11–13], the bradykinin concentration may rise and favor the development of angioedema [2, 14–15].

Environmental factors that are reported to increase the risk of angioedema occurrence include co-medications such as acetylsalicyclic acid or non-steroidal anti-inflammatory drugs (NSAID), immunosuppressive agents used in transplant patients, DPPIV inhibitors (DPPIVi), fibrinolytics and estrogens [14, 16, 17, 18]. In addition, female gender (relative risk RR: 1.45, 95%-CI: 0.82–0.95) [5, 19] and smoking have been identified as risk factors for ACEi-associated angioedemas (hazard ratio [HR]: 2.7, 95%-CI: 1.1–7.0) [20, 21].

Concerning the genetic association, on a more general basis, Afro-American descent is described to increase the risk ([RR]: 3.88, 95%-KI: 2.99–4.95) [1, 5, 19, 20]. On a more detailed level, genetic variants that affect the ACEi gene function or the bradykinin receptors, as well as genes involved in fibrinolytic and coagulation or immune response and inflammatory pathways have been identified as risk factors. However, the results of these genetic associations were not strong and have not been replicated, so far [22].

The angioedema incidence for angiotensin-receptor blockers (ARBs) is reported to be lower [8, 23], than for ACEi. For aliskiren (renin inhibitor) lower [23] and equal angioedema incidences [8, 24] are reported compared to ACEi.

ARBs, as well as aliskiren, do not interact with ACE directly [25, 24, 26] and should therefore not affect bradykinin levels through this pathway. For ARBs and aliskiren the pathophysiology causing an angioedema is not fully understood [26, 27]. To date, literature is inconsistent as to whether ARBs, and/or aliskiren can be used as an alternative treatment after ACEi-associated angioedema occurred [27–29].

ACEi therapy is recommended as one of the first-line treatments for hypertension and heart failure in national and international guidelines [30, 31]. Therefore, the worldwide number of patients exposed to ACEi is huge [9]. In Germany, an enormous increase of ACEi prescriptions has been observed over the past few years [32]. A national study evaluated that ACEi was the drug class most frequently taken in 2008–2011, with a significantly higher use in males than females [33]. In contrast, ARBs and aliskiren are prescribed much less frequently than ACEi. However, ARB prescriptions have increased during the time frame of our analysis (2000–2016) [32].

To the best of our knowledge, this is the first retrospective comparative analysis of angioedema reports associated with ACEi, ARBs and aliskiren performed in the European adverse drug reaction database EudraVigilance (analyzing tool: EVDAS) of the European Medicine Agency (EMA) [34] and the national ADR database of the Federal Institute for Drugs and Medical Devices (BfArM) [35] in Germany. The first aim of the present study was to analyze whether there are characteristics more often reported in ACEi, ARBs and aliskiren angioedema reports compared to their respective controls. The second aim was to analyze whether there are differences between ARBs and aliskiren versus ACEi angioedema reports concerning the reported characteristics and clinical phenotypes. The third aim was to analyze the differences between the high-level analyses in **EVDAS** covering the entire European Economic Area (EEA) versus the analysis of national validated cases of **BfArM’s ADR-database**.

This topic is highly relevant due to the high and increasing number of patients exposed to RASi which may lead to an increase of potentially life-threatening angioedemas.

## Materials and methods

### 1.) *BfArM’s ADR-database* and *EVDAS*

Physicians in Germany are obliged by their professional conduct code to report ADRs to their professional councils. These forward the reports to either the Federal institute for Drugs and Medical Devices (BfArM) [35] (responsible for chemically defined drugs) or the Paul-Ehrlich-Institut (PEI) [36] (responsible for monoclonal antibodies, vaccines etc.), as described elsewhere [37, 38]. Physicians may also have reported directly to marketing authorization holders. All reports received up until 22/11/2017 were stored in one of the two national **ADR-databases** in accordance with the responsibilities of the aforementioned competent authorities and forwarded to **EudraVigilance**, the database of the European Medicines Agency (EMA) [39]. However, on 22/11/2017 both national **ADR-databases** were closed and since then marketing authorization holders as well as the national competent authorities report serious and non-serious ADRs directly to the EMA [39].

In the presented study we performed two separate analyses. The analysis covering the entire European Economic Area (EEA) was performed in **EVDAS**. **EVDAS** is the interface for analyzing ADR reports in **EudraVigilance** [40]. The analysis of the national ADR reports (originating from Germany) was performed in a validated dataset (see 2.2.1.) of **BfArM’s ADR-database**.

In **BfArM’s ADR-database**, drugs are coded in accordance with the Drug Dictionary of the World Health Organization (WHO) [41] and the Anatomical Therapeutic Chemical (ATC) classification system [42]. In **EVDAS**, drugs are coded in accordance with the EudraVigilance medicinal product dictionary (XEVMPD or Article 57 database) [43]. ADRs are coded in accordance with the terminology of the Medical Dictionary for Regulatory Activities (MedDRA) [44] in both databases. The MedDRA terminology includes five different hierarchical levels for coding, and thus for the analysis of the ADRs reported. The highest level of the MedDRA terminology enables an analysis of aggregated data (coarse-grained data) with lowest specificity. In contrast, the lowest level of the MedDRA terminology enables a finer-grained analysis with highest specificity. The most specific level is designated as "Lowest Level Term (LLT)" and represents the ADR/s reported in clinical practice. Each LLT belongs to one preferred term (PT). Each PT summarizes the LLTs and describes the symptom, investigation or disease diagnosis. These PTs are assigned to the High Level Terms (HLTs) and High Level Group Terms (HLGTs) based on their anatomy, pathology, physiology, etiology or function. The HLGTs are assigned to the System Organ classes (SOCs). The SOCs represent the anatomical areas in which the ADR occurs and are, thus, the aggregated level of analysis.

### 2.) Identification of cases in *EVDAS* and *BfArM’s ADR-database*

#### 2.1) *EVDAS*

**Fig 1:**
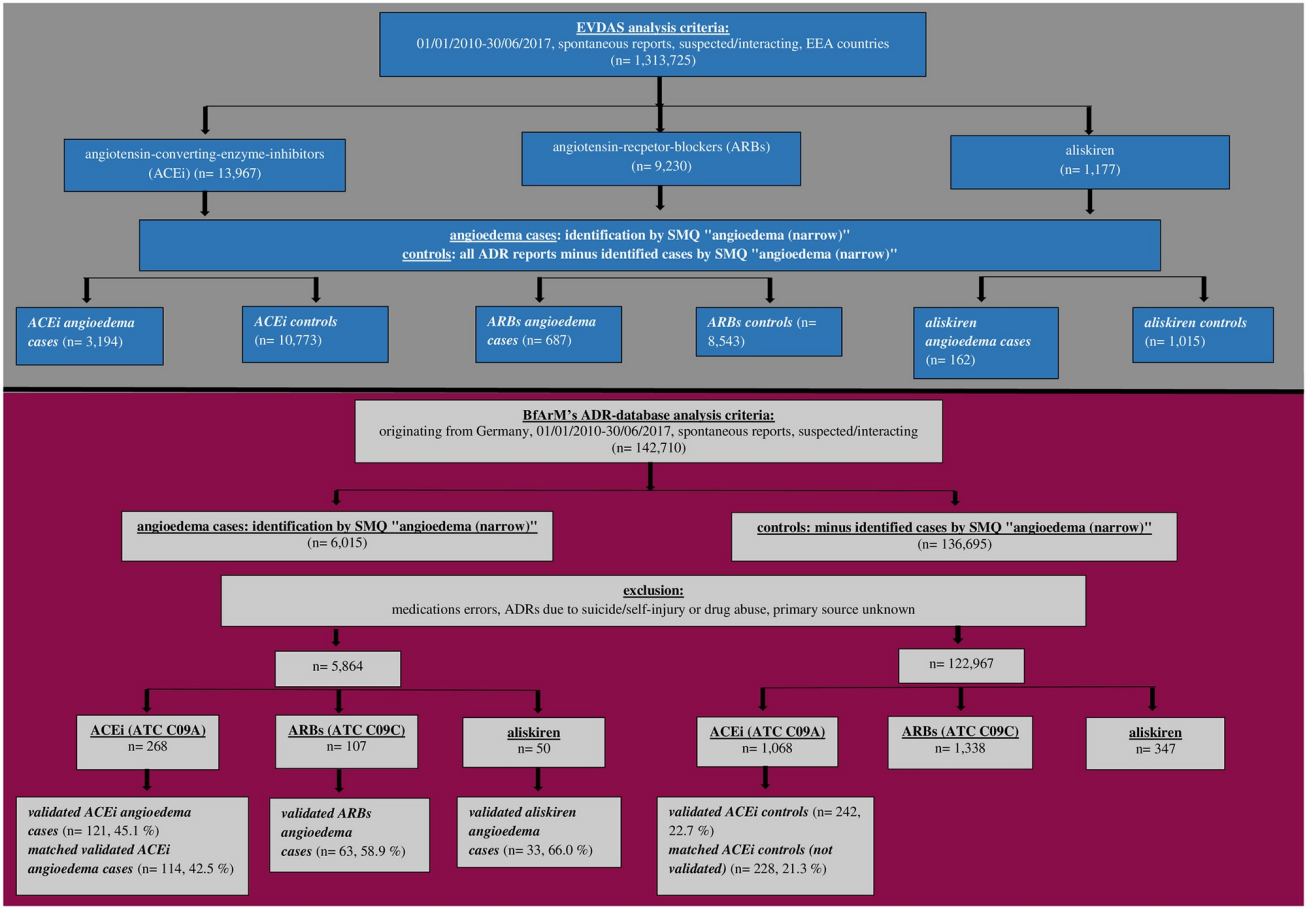
Flowchart. Fig 1 represents the number of cases identified for *ACEi*, *ARBs* and *aliskiren angioedema cases* and their respective *controls* in **EVDAS** and **BfArM’s ADR-database.**

In **EVDAS**, all spontaneous ADR reports registered between 01/2010 and 06/2017 within the EEA were identified in which either an ACEi, ARB or aliskiren was reported as a "suspected/interacting" drug monosubstance (query date: 17/12/2018) (Fig 1)). For each RASi, the angioedema cases were extracted by application of the standardized MedDRA Query (SMQ) "angioedema (narrow)" [45]. A SMQ is a validated standard set of specific and less specific MedDRA terms at the PT level that facilitates the retrieval of MedDRA coded data. In order to identify specific or specific and less specific terms, one can choose among a narrow and a broad search strategy. These differ in their specificity and sensitivity. Narrow searches are used to identify symptoms that are highly likely to represent the condition of interest. In contrast, broad searches also include symptoms and signs with little or no interest on closer inspection. For the present analysis we chose the narrow search in order to identify ADRs that are more likely representative for angioedemas.

In addition, for each drug class a dataset of controls was generated consisting of all other ADR reports excluding *angioedema cases*.

#### 2.2) *BfArM’s ADR-database*

For the analysis in **BfARM’s ADR-database** the same research strategy as applied in **EVDAS** was used for the identification of *ACEi*, *ARBs and aliskiren angioedema cases*. Deviating from the **EVDAS** analysis, we restricted our dataset to ADRs that occurred in association with the intended drug use. Therefore, we excluded all ADR reports in which a medication error or drug intake due to intentional suicidal/self-injury behaviors was described by application of respective SMQs. Furthermore, we excluded ADR reports with unknown sender.

*2*.*2*.*1) BfArM’s ADR-database*: *Validation of angioedema cases*. In order to strengthen the results of the high-level **EVDAS** analysis and to broaden the analysis with information provided in more detail in the case narratives (e.g. treatment of angioedema), an assessment of each individual RASi angioedema report with German origin was performed by the author DD. The causal relationship with the reported "suspected/interacting" RASi was assessed according to WHO criteria [46]. Those reports for which the causal relationship was assessed as at least "possible" were subjected to further analysis. Additionally, the correctness of the diagnosis "angioedema" was assessed. Therefore, all angioedema cases were reviewed in detail to confirm that swellings/oedemas of the head areas (e.g. lips, face), the respiratory tract (e.g. tongue, pharynx), the intestinal tract or genitals were reported. Some reports only provided the diagnosis "angioedema". These reports were only considered for further analysis if angioedema treatment was in accordance with medical practice and led to symptom relief or if a physician reported the diagnosis "angioedema" based on the assumption of existing medical expertise. We excluded all reports in which the angioedema was more likely induced by other causes, e.g. heart failure, tooth extractions. Reports that could not be unambiguously assigned with regard to causality or the correctness of the diagnosis were discussed together by the authors DD and BS prior to the final assignment.

*2*.*2*.*2) BfArM’s ADR-database*: *Generation of validated ACEi controls*. In order to establish a dataset of *validated ACEi controls* in a 2:1 ratio to the *validated ACEi angioedema cases* (n = 121), a random sample of the identified *ACEi controls* (n = 1,068) was selected. This random sample was assessed with regard to the causal relationship as described above until 242 *validated ACEi controls* were available. The ADRs reported most often in the *validated ACEi controls* were "cough" (17.8%), "acute kidney injury" (9.9%), "dizziness" (9.1%), "nausea" (5.0%) and "hyperkaliaemia" (4.5%).

Additionally a 1:2 matching by age and gender of *validated ACEi angioedema cases* to *ACEi controls* (not validated, n = 1,068) was performed in order to confirm the observed results between *validated ACEi angioedema cases* versus *validated controls*. In seven *validated ACEi angioedema cases*, the age or gender of the patient was missing. Thus, the datasets of *matched validated ACEi angioedema cases* and *controls* include 114 and 228 cases.

*2*.*2*.*3) BfArM’s ADR-database*: *Documentation quality of validated cases*. Finally, the quality (completeness of reports) of all *validated angioedema cases* and the *validated ACEi controls* was assessed according to a published score (vigiGrade) [47]. The calculation of the score was modified as it was computed for the reported diagnosis "angioedema", only [48].

### 3.) *EVDAS* and *BfArM’s ADR-database*: Analysis of angioedema cases and controls

In both databases, all identified angioedema cases and controls were analyzed with regard to the reported patient demographics, smoking habits, comorbidities, administered ACEi (for reports of ACEi), ARBs (for reports of ARBs), comedications and the reported seriousness criteria. Gender-stratified analyses were performed in *ACEi angioedema cases*.

Comparative analyses were conducted between *ACEi*, *ARBs*, *aliskiren angioedema cases* versus their respective *controls*, and between *ACEi angioedema cases* versus *ARBs* and *aliskiren angioedema cases*, separately.

All analyses in **EVDAS** were computer-based without individual assessment of the cases. Smoking, allergic conditions and comorbidities were identified by summarizing appropriate preferred terms [44] or by application of appropriate SMQs [45].

Any analysis in **BfArM’s ADR-database** was based on the information provided in the complete report including narrative and follow-ups.

The classes of comedications were formed in accordance with the ATC-code [42]. Therefore, all drugs co-reported to the "suspected/interacting" RASi were assessed as concomitant, regardless of whether they had been reported as "suspected", "interacting" or "concomitant". Furthermore, the analysis of comedications was restricted to the drugs most frequently reported and/or reported in literature to potentiate the risk of angioedema occurrence when used concomitantly with ACEi (e.g. (DPPIVi or mammalian target of rapamycin (mTOR) inhibitors (mTORi)) [16, 17, 18].

According to the legal definition, an ADR is considered serious if it led to "death", was "life-threatening", required or prolonged "hospitalization", resulted in persistent or significant "disabilities" and/or was a "congenital anomaly/birth defect" [38]. Hence, this classification of seriousness of the ADR report may differ from the clinical severity of the ADR.

The number of reports per anatomical area affected by the angioedema was analyzed in **EVDAS** for all three RASi, and for *ACEi angioedema cases* with concurrent mTORi, firbinolytics, and DPPIVi therapy. It should be pointed out that mTORi, fibrinolytics and DPPIVi themselves are also associated with angioedemas.

In order to investigate angioedemas that are probably related to the respective RASi, the analysis was restricted to reports in which only the respective RASi was reported as "suspected" (exclusion of cases in which other drugs had been reported as co-suspected). Hence, 77.3% (2,469/3,194) of *ACEi*, 71.5% (491/687) of *ARBs* and 82.7% (134/162) of *aliskiren angioedema cases* remained.

Concerning these remaining cases, in 54.9% (1,355/2,469) of *ACEi*, 41.8% (205/491) of *ARBs* and 32.8% (44/134) of *aliskiren angioedema cases*, only the diagnosis "angioedema" was reported. Since information about the affected anatomical areas may be reported in the narratives of the cases, the same analysis was repeated in the analysis of **BfArM’s ADR-database.** Further on, in these validated cases a stratified analysis of anatomical areas affected by the angioedema was conducted.

The aforementioned analyses were also conducted for sacubitril/valsartan. Due to the limited number of cases, the results were not included in the manuscript (S1 File).

#### 3.2) *BfArM’s ADR-database* analysis

*3*.*2*.*1*.*) Number of ADR reports in relation to the number of assumed ACEi-exposed inhabitants/males/females*. The number of assumed ACEi-exposed inhabitants/males/females was estimated based on the number of inhabitants/males/females per year [49] multiplied by the proportional share of ACEi exposure in the German population (DEGS1) [33]. The average and its standard deviation (+/-SD) of the number of angioedema and ADR reports (total) divided by the number of assumed ACEi-exposed inhabitants/males/females for the six years was calculated. The results are presented as the number of ADR reports per 1 million assumed ACEi-exposed inhabitants/males/females. Unfortunately, the proportional share of ARBs and aliskiren exposure in the German population was not reported in DEGS1. Thus, this calculation could not be performed for ARBs and aliskiren.

**Fig 2:**
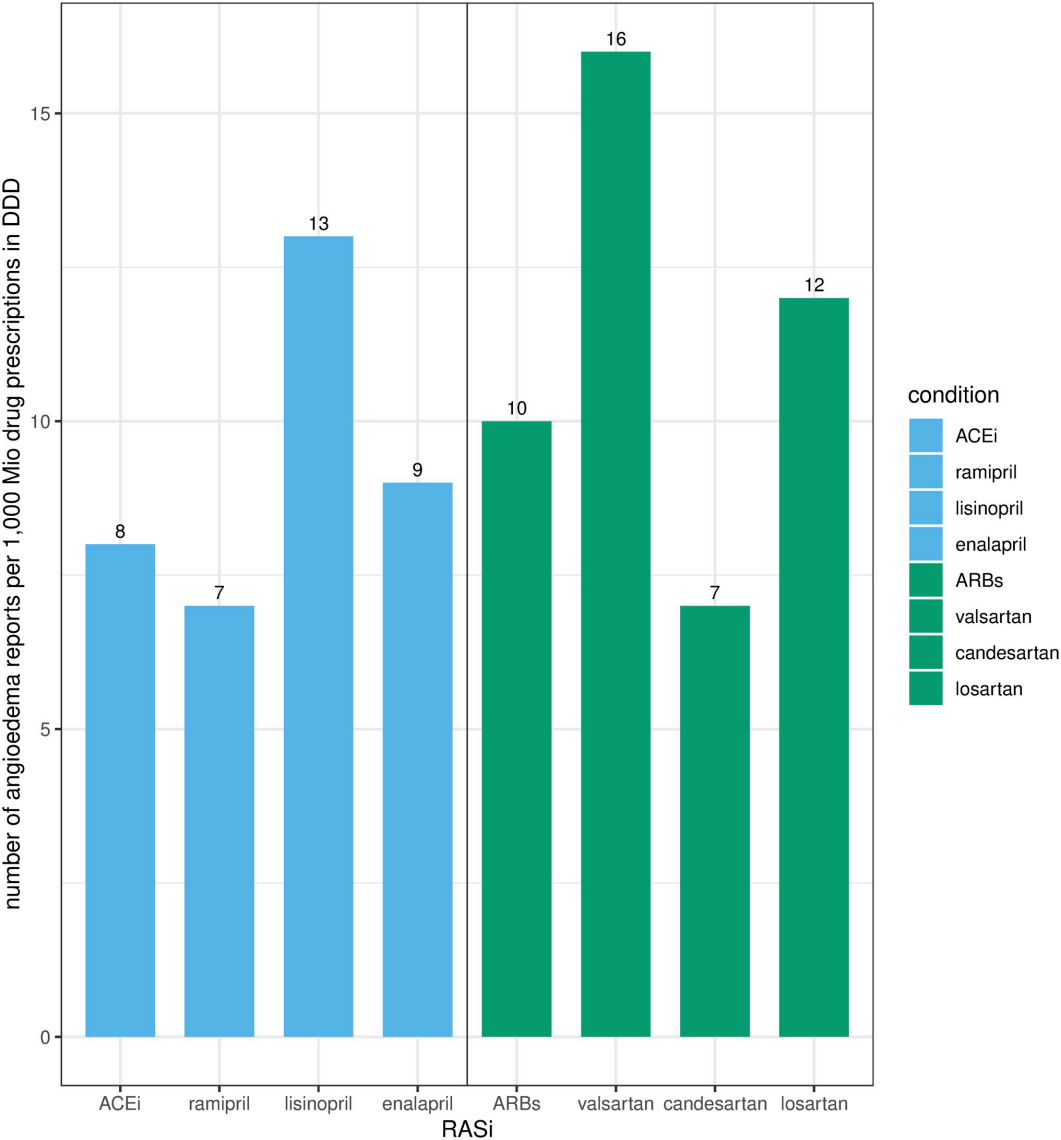
Number of *ACEi*, *ARBs*, and *aliskiren angioedema cases* per 1,000 Mio drug prescriptions in DDD (2010–2016). Fig 2 shows the number of angioedema reports per 1,000 Mio drug prescriptions in DDD for ACEi, and ARBs. For aliskiren, 154 angioedema reports per 1,000 Mio drug prescriptions were calculated. The number is not depicted in Fig 2 in order to make the difference between the respective drug substances of ACEi and ARBs clearer. The complete presentation of the number of cases and the number of drug prescriptions used for the calculation of the number of angioedema reports per 1,000 Mio drug prescriptions in DDD is contained in S6 Table. Our result deviates from existing literature. With regard to a meta-analysis of randomized trials for renin-angiotensin system inhibitors associated angioedemas, the incidences for ACEi were 0.30% for ARBs 0.11% and for aliskiren 0.13% [23]. The limitations of spontaneous reporting systems have to be considered.

*3*.*2*.*2*.*) Number of ADR reports in relation to the number of drug prescriptions*. Annually published prescription data (Drug Prescription Reports 2011–2017) [32] were used to summarize the number of drug prescriptions (in million DDD) for ACEi, ARBs and aliskiren monosubstances for the years 2010–2016 in Germany. Hence, the time frame of **BfArM’s ADR-database** analysis had to be adapted to 01/2010-12/2016. The average (+/-SD) of the number of angioedema and ADR reports (total) divided by the number of drug prescriptions for the six years was calculated. The drug prescription reports contain the number of drug prescriptions in defined daily doses (DDD) [32]. However, the DDD may deviate from the administered or prescribed dose to a varying extent depending on the individual drug [50]. Therefore, angioedema incidence rates observed in a meta-analysis of clinical trials are also described in the legend of Fig 2 and depicted in S6 Table [23].

*3*.*2*.*3) Additional analysis*: *Time-to-onset and treatment of angioedema*. Both "time-to-onset" (i.e. time point of first intake of the suspected drug to time point of first onset of the ADR) and the treatment of the angioedema, including its clinical response, are often described in more detail in the narratives of the ADR reports. Hence, these analyses were only performed in validated cases.

### 4.) Statistical analysis

Mean and median were calculated for the age of the patients and frequency distribution for all other variables. Odds ratios (ORs) and the 95% confidence interval (CI) were calculated in order to assess differences in the frequency distributions between the compared groups.

A logistic regression analysis was performed for each comparison of *angioedema cases* versus controls, and *ACEi angioedema cases* versus *ARBs* and *aliskiren angioedema cases* as outcome variable and all other variables (if possible) as covariates. Diabetes was not included as a variable in the logistic regression model to avoid overlaps with the variable "antidiabetics". The same applies to the variables "death", "life-threatening", "hospitalization" and "disabling" with regard to the variable "serious" (the definition "serious" includes all of the aforementioned variables). Results obtained from logistic regression are reported in terms of OR with 95% CI. In logistic regression analysis, the age of the patients was stratified in patients 65 years and older versus patients younger than 65 years.

In **BfArM’s ADR-database** analysis, a sensitivity analysis by multiple imputation using the MICE package for R version 3.5.2 was performed for comparison of *validated ACEi angioedema cases* and *validated ACEi controls*, since 22 cases were incomplete (gender was unknown in two cases, age was unknown in 21 cases, both variables were missing in one case).

The ADR reports are included in the databases in a pseudonymized form. In accordance with the formal requirements, the reporting of ADRs in the post-marketing setting does not require any consent from the patient affected by the ADR. The study had been approved by the local ethics committee of the Medical Faculty of Bonn (009/17). Since the closure of BfArM’s ADR-database, public access to the restricted set of data elements is no longer available. Due to data privacy requirements, it is not possible to make the complete individual case report available to the readership [51]. Researchers and/or readers who are interested can perform the same analysis in the ADR database EudraVigilance of the EMA (public access: http://www.adrreports.eu/en/index.html). However, different levels of access are granted for different stakeholders [52]. Nevertheless, even with the lowest level of access an analysis of aggregated data is possible.

## Results

### 1.) Summary of reported characteristics in RASi angioedema cases and controls

#### 1.1) *EVDAS* analysis

The age and gender distribution of *ACEi angioedema* cases and *controls* was almost equal (Table 1). Histories of "allergy" (OR 1.8 [1.4–2.3]), "previous/recurrent angioedema" (OR 36.8 [18.5–73.3]) or "urticaria" (OR 3.5 [1.4–8.4]) and "asthma" (OR 1.7 [1.2–2.3]) were reported more often in *ACEi angioedema cases* than in controls. In contrast, "renal disorders" (OR 0.6 [0.5–0.8]) were reported more frequently in *ACEi controls*. Enalapril (OR 1.9 [1.6–2.3]) and lisinopril (OR 2.0 [1.6–2.5]) had been administered more often in *ACEi angioedema cases* than in controls. Likewise, mTORi (OR 8.9 [4.9–16.4]) and fibrinolytics (mostly alteplase) (OR 16.3 [7.5–35.1]) had been used as concomitant medication more frequently in *ACEi angioedema cases* than in *controls*.

**Table 1:** *EVDAS* analysis: Reported characteristics in *ACEi angioedema cases* and *ACEi controls* and comparative analysis of *ACEi angioedema cases* versus *ARBs* and *aliskiren angioedema cases*.

*EVDAS* analysis	Characteristics *ACEi angioedema cases* and *ACEi controls*		*ACEi angioedema cases* versus *ACEi controls*		*ACEi angioedema cases* versus *ARBs angioedema cases* ^k^		*ACEi angioedema cases* versus *aliskiren angioedema cases* ^l^	
	*ACEi angioedema cases* (n = 3,194; 22.9%)	*ACEi controls* (n = 10,773; 77.1%)	unadjusted OR [+/- 95% CI]	logistic regression OR [+/- 95% CI]	unadjusted OR [+/- 95% CI]	logistic regression OR [+/- 95% CI]	unadjusted OR [+/- 95% CI]	logistic regression OR [+/- 95% CI]
***patient demographics***								
mean age (median) [years] ^a^	66.8 (68.0)	67.1 (69.0)	-	1.0 [0.9–1.1]	-	1.0 [0.9–1.2]	-	1.1 [0.7–1.6]
female	47.2% (1,506)	48.6% (5,239)	0.9 [0.9–1.0]	1.0 [0.9–1.1]	0.6 [0.5–0.8]*	0.7 [0.6–0.9]*	0.5 [0.4–0.8]*	0.6 [0.4–0.9]*
male								
unknown	50.6% (1,617)	49.4% (5,323)						
	2.2% (71)	2.0% (211)						
***patients history***								
smoker ^b^	2.1% (66)	2.4% (255)	0.9 [0.7–1.1]	0.8 [0.6–1.1]	1.0 [0.6–1.9]	0.8 [0.4–1.5]	1.6 [0.4–6.7]	1.3 [0.3–5.7]
allergy ^c^	4.3% (137)	2.1% (228)	2.1 [1.7–2.6]*	1.8 [1.4–2.3]*	0.6 [0.4–0.9]*	0.8 [0.5–1.1]	0.3 [0.5–0.2]*	0.4 [0.2–0.8]*
urticaria	0.5% (17)	0.1% (9)	6.4 [2.8–14.4]*	3.5 [1.4–8.4]*	0.5 [0.2–1.2]	0.5 [0.2–1.3]	-	-
angioedema ^d^	4.0% (129)	0.1% (9)	50.3 [25.6–99.1]*	36.8 [18.5–73.3]*	1.1 [0.7–1.9]	1.1 [0.7–1.8]	1.0 [0.4–2.4]	1.4 [0.5–3.8]
***comorbidities*** ^e^								
renal disorders	4.5% (144)	6.4% (694)	0.7 [0.6–0.8]*	0.6 [0.5–0.8]*	2.5 [1.4–4.5]*	1.9 [1.1–3.5]*	1.2 [0.5–2.7]	0.9 [0.2–2.2]
diabetes	10.2% (325)	11.2% (1,206)	0.9 [0.8–1.0]	-	1.4 [1.0–1.9]	-	0.9 [0.5–1.4]	-
asthma	2.3% (74)	1.3% (137)	1.8 [1.4–2.4]*	1.7 [1.2–2.3]*	0.9 [0.5–1.6]	0.9 [0.5–1.7]	0.4 [0.2–0.9]*	0.5 [0.2–1.1]
malignant tumors	4.0% (127)	4.3% (462)	0.9 [0.8–1.1]	0.9 [0.7–1.1]	1.4 [0.8–2.2]	1.1 [0.7–1.8]	1.6 [0.6–4.3]	1.8 [0.5–5.9]
thyroid disorders	2.6% (82)	2.8% (306)	0.9 [0.7–1.2]	0.9 [0.7–1.2]	0.8 [0.5–1.3]	0.8 [0.5–1.3]	0.6 [0.3–1.2]	0.6 [0.3–1.5]
***administered ACEi*** ^f^								
ramipril	37.4% (1,195)	45.3% (4,884)	0.7 [0.7–0.8]*	1.2 [1.0–1.4]	-	-	-	-
enalapril	28.2% (902)	21.8% (2,346)	1.4 [1.3–1.5]*	1.9 [1.6–2.3]*	-	-	-	-
perindopril	16.1% (514)	15.6% (1,676)	1.0 [0.9–1.2]	1.4 [1.2–1.7]*	-	-	-	-
lisinopril	13.1% (419)	10.2% (1,096)	1.3 [1.2–1.5]*	2.0 [1.6–2.5]*	-	-	-	-
***comedication*** ^g^								
β-blockers	22.7% (725)	30.3% (3,259)	0.7 [0.6–0.7]*	0.8 [0.7–0.9]*	1.4 [1.1–1.7]*	1.1 [0.9–1.4]	1.3 [0.9–2.0]	1.1 [0.7–1.9]
diuretics	21.9% (700)	34.5% (3,715)	0.5 [0.5–0.6]*	0.5 [0.4–0.6]*	1.2 [0.9–1.4]	1.0 [0.8–1.2]	0.9 [0.6–1.3]	0.8 [0.5–1.2]
calcium antagonists	17.5% (558)	16.9% (1,817)	1.0 [0.9–1.2]	1.1 [1.0–1.2]	1.4 [1.1–1.9]*	1.1 [0.8–1.4]	0.6 [0.4–0.8]	0.4 [0.3–0.6]*
ARBs	4.0% (127)	4.8% (518)	0.8 [0.7–1.0]	0.8 [0.7–1.0]	-	-		
acetylsalicyclic acid	19.9% (636)	20.7% (2,235)	0.9 [0.9–1.0]	1.1 [1.0–1.2]	2.0 [1.5–2.5]*	1.4 [1.1–1.8]*	1.6 [1.0–2.5]	1.5 [0.9–2.6]
analgesics ^h^	11.4% (365)	13.6% (1,469)	0.8 [0.7–0.9]*	0.8 [0.7–0.9]*	1.3 [1.0–1.8]*	1.1 [0.8–1.6]	1.9 [1.0–3.6]	2.6 [1.2–5.7]*
antidiabetics ^i^	10.1% (322)	12.8% (1,376)	0.8 [0.7–0.9]*	0.8 [0.7–0.9]*	1.0 [0.7–1.3]	1.2[0.9–1.8]	1.2 [0.7–2.0]	1.6 [0.8–3.2]
DPPIVi	2.1% (67)	2.2% (232)	1.0 [0.7–1.3]	0.9 [0.6–1.2]	0.7 [0.4–1.1]	0.5 [0.3–0.9]*	0.5 [0.2–1.3]	0.6 [0.2–1.8]
mTORi	1.3% (42)	0.2% (18)	8.0 [4.6–13.8]*	8.9 [4.9–16.4]*	4.3 [1.0–17.9]	2.8 [0.7–12.0]	-	-
fibrinolytics	1.2% (38)	0.1% (9)	14.4 [7.0–29.8]*	16.3 [7.5–35.1]*	7.8 [1.1–57.2]*	-	-	-
***seriousness criteria*** ^j^								
serious	88.8% (2,836)	73.9% (7,965)	2.8 [2.5–3.1]*	3.3 [2.9–3.7]*	1.8 [1.5–2.3]*	1.8 [1.4–2.3]*	0.3 [0.1–0.6]*	0.3 [0.1–0.9]*
death	1.6% (52)	2.7% (288)	0.6 [0.4–0.8]*	-	2.7 [1.0–7.4]	-	0.6 [0.2–1.8]	-
life-threatening	15.5% (496)	5.9% (632)	2.9 [2.6–3.3]*	-	2.2 [1.6–2.9]*	-	14.2 [3.5–57.4]*	-
hospitalization	50.7% (1,619)	45.6% (4,909)	1.2 [1.1–1.3]*	-	2.3 [1.9–2.8]*	-	5.4 [3.5–8.3]*	-
disabling	0.8% (27)	2.4% (262)	0.3 [0.2–0.5]*	-	0.3 [0.2–0.5]*	-	0.7 [0.2–2.8]	-

*OR = 1 is not included; OR > 1 reported more often in *ACEi angioedema cases*; OR < 1 reported more often in *ACEi controls*, *ARBs angioedema cases*, *alisiren angioedema cases*

^a^ age unknown: *ACEi angioedema cases*: 179 cases (5.4% of cases), *ACEi controls*: 717 cases (6.7% of cases).

^b^ refers to current smoking at the time of the reported ADR. Former smokers were classified as non-smokers.

^c^ the term "allergy" refers to a reported allergy and the occurrence of any allergic and hypersensitivity reactions reported in the history of the patient.

^d^ urticaria was analyzed based on the HLT "urticarias". The term "angioedema" summarizes previous angioedema or swellings coded in the SMQ "angioedema (narrow)" reported in the history of the patient.

^e^ suitable hierarchical levels of the MedDRA terminology were chosen for analysis of the reported patients’ comorbidities. The term "renal disorders" was identified using the SMQs "acute renal failure" and "chronic kidney disease"; "diabetes": SMQ "hyperglycaemia/new onset diabetes mellitus"; "asthma": SMQ "asthma/bronchospasm"; "malignant tumors": SMQ "malignant tumours"; "thyroid disorders": SMQ "thyroid dysfunction".

^f^ the four ACEi monosubstances most frequently reported as "suspected/interacting" are tabulated. The relative number of ADR reports specifying one of the remaining ACEi (not listed) as "suspected/interacting" was lower than 2%. One ADR report may contain more than one ACEi as "suspected/interacting" drug substance. Thus, the number of reported ACEi exceeds that of the ADR reports.

^g^ the analysis of the most frequently reported and most relevant comedications refers to monosubstances and combination products of the tabulated drug substances and/or drug classes and corresponds to the ATC classification. All drugs co-reported to the "suspected/interacting" ACEi were assessed as concomitant, regardless of whether they had been reported as "suspected", "interacting" or "concomitant".

^h^ deviating from the ATC-code, the analysis concerning "analgesics" also includes ADR reports in which ibuprofen and/or diclofenac were listed as suspected/interacting or concomitant drug. We excluded ADR reports in which acetylsalicyclic acid was listed as suspected/interacting or concomitant drug. The number of ADR reports in which acetylsalicyclic acid was used concurrently were analyzed separately.

^i^ deviating from the ATC-code, we excluded ADR reports in which a DPPIVi was listed as suspected/interacting or concomitant drug in the analysis concerning "diabetics". The number of ADR reports in which DPPIVi was used concurrently was analyzed separately.

^j^ one ADR report may yield information about more than one seriousness criterion, therefore, the number of reported seriousness criteria exceeds that of the ADR reports.

^k^ 44 cases which were included in *ACEi angioedema cases* and *ARBs angioedema cases* were excluded.

^l^ 6 cases which were included in *ACEi angioedema cases* and *aliskiren angioedema cases* were excluded.

Table 1 shows the absolute and relative number of reports for the reported demographic parameters, comorbidities, comedications and seriousness criteria of *ACEi angioedema cases* and ACEi *controls* and the calculated unadjusted and adjusted odds ratios of *ACEi angioedema cases* versus *ACEi controls*, versus *ARBs angioedema cases* and versus *aliskiren angioedema cases*. The raw data of the *ARBs* and *aliskiren angioedema cases* as well as their unadjusted and adjusted odds ratio compared to their *controls* are presented in S2 Table.

Gender-stratified analysis of *ACEi angioedema* cases revealed that a previous history of "allergy" (OR 2.3 [1.6–3.4], "urticaria" (OR 3.0 [1.0–9.2]), "asthma" (OR 1.8 [1.1–3.1]), "thyroid disorders" (OR 5.6 [3.1–10.0]) as well as concurrent use of diuretics (OR 1.5 [1.2–1.8]) and analgesics (OR 1.3 [1.1–1.7]) was more often reported for females than for males (S1 Table). In contrast, being a smoker (OR 0.3 [0.2–0.6]) and having a history of "previous/recurrent angioedema" (OR 0.5 [0.3–0.7]), "renal disorders" (OR 0.5 [0.4–0.8]) and concurrent treatment with a calcium antagonist (OR 0.8 [0.6–0.9]) and acetylsalicyclic acid (OR0.6 [0.5–0.8]) were more frequently reported for males than for females.

*ACEi angioedema cases* were more frequently designated as "serious" and "life-threatening" than *ACEi controls* (Table 1). Half of the *ACEi angioedema case* (50.7%) either led to or prolonged "hospitalization".

Almost the same observations (but with different frequencies as seen in *ACEi angioedema cases*) were noted for *ARBs* and *aliskiren angioedema cases* with regard to reported "allergy", "previous/recurrent angioedema" and comorbidities when compared to their controls (S2 Table). More females in *aliskiren angioedema cases* (OR 1.5 [1.0–2.2]), a higher concomitant drug use of DPPIVi in *ARBs* (OR 1.8 [1.1–3.1]) and *aliskiren angioedema cases* (OR 1.6 [0.5–5.2]) as well as concurrent ACEi use in *ARBs angioedema cases* (OR 2.2 [1.6–3.0]) were observed compared to their respective controls.

#### 1.2) *BfArM’s ADR-database* analysis

Slightly more males (53.7% versus females 43.4%) were included in the *validated ACEi angioedema cases* versus *validated ACEi controls* of **BfArM’s ADR-database** analysis (Table 2). However, after relating the number of ACEi angioedema reports to the assumed ACEi-exposed inhabitants/males/females (DEGS1) [33], ACEi-associated angioedema cases referred 1.5 times more often to females than to males (S3 Table).

**Table 2:** *BfArM’s ADR-database* analysis: Characteristics of *validated ACEi angioedema cases* and *validated ACEi controls*.

*BfArM’s ADR-database* analysis	*characteristics of validated ACEi angioedema cases and validated controls*		*validated ACEi angioedema cases* versus *validated ACEi controls*			
	*validated ACEi angioedema cases* (n = 121)	*validated ACEi controls* (n = 242)	unadjusted OR [+/- 95% CI]	logistic regression OR [+/- 95% CI]	logistic regression p-values	logistic regression + imputation (MICE) p-values
***completeness score*** ^a^	0.74 [0.65–0.82]	0.71 [0.65–0.77]	-	-	-	-
***patient demographics*** ^b^						
mean age (median)	64.5 (68)	63.5 (65)	-	1.5 [0.9–2.7]´	0.121	0.099
[years]			-			
female	46.3% (56)	55.8% (135)	0.7 [0.4–1.0]	0.9 [0.5–1.5]	0.569	0.665
male	53.7% (65)	% (105)				
***smoking and drinking***						
*habits*, *allergic*						
*conditions*						
smoker ^c^	14.0% (17)	3.3% (8)	4.8 [2.0–11.4]*	2.7 [1.0–7.6]	0.058	0.043*
alcohol consumption ^d^	9.1% (11)	2.5% (6)	3.9 [1.4–10.9]*	2.9 [0.8–10.4]	0.098	0.088
allergy ^e^	12.4% (15)	10.3% (25)	1.2 [0.6–2.4]	1.0 [0.5–2.3]	0.942	0.988
angioedema ^f^	24.0% (29)	-	-	-	-	-
***comorbidities*** ^g^						
renal disorders	9.9% (12)	8.7% (21)	1.2 [0.5–2.4]	1.0 [0.4–2.3]	0.953	0.749
diabetes	15.7% (19)	13.2% (32)	1.2 [0.7–2.3]	1.1 [0.5–2.2]	0.892	0.951
asthma/COPD	9.1% (11)	6.2% (15)	1.5 [0.7–3.4]	1.8 [0.7–4.8]	0.253	0.231
***administered ACEi*** ^h^						
ramipril	67.8% (82)	75.2% (182)	0.7 [0.4–1.1]	1.4 [0.4–5.0]	0.620	0.997
enalapril	16.5% (20)	12.4% (30)	1.4 [0.8–2.6]	1.4 [0.4–5.8]	0.607	0.822
lisinopril	10.7% (13)	9.1% (22)	1.2 [0.6–2.5]	1.5 [0.4–6.7]	0.563	0.770
***comedication*** ^i^						
β-Blocker	28.1% (34)	23.1% (56)	1.3 [0.8–2.1]	1.6 [0.8–3.0]	0.165	0.275
diuretics	13.2% (16)	17.4% (42)	0.7 [0.4–1.4]	0.4 [0.2–0.8]*	0.023*	0.023*
calcium antagonists	17.4% (21)	9.1% (22)	2.1 [1.1–4.0]*	1.6 [0.7–3.3]	0.248	0.181
NSAID	21.5% (26)	19.8% (48)	1.1 [0.6–1.9]	0.5 [0.3–1.0]	0.057	0.083
everolimus	5.8% (7)	0.0% (0)	-	-	-	-
alteplase	0.8% (1)	0.0% (0)	-	-	-	-
***seriousness criteria*** ^j^						
serious	89.3% (108)	53.7% (130)	7.2 [3.8–13.4]*	7.7 [3.9–15.1]*	< 0.001*	< 0.001*
death	3.3% (4)	1.2% (3)	2.7 [0.6–12.4]	-	-	-
life-threatening	28.9% (35)	5.0% (12)	2.8 [1.6–4.8]*	-	-	-
hospitalization	49.6% (60)	28.5% (69)	2.5 [1.6–3.9]*	-	-	-
disabling	0.8% (1)	5.0% (12)	0.2 [0.0–1.2]	-	-	-

*OR = 1 is not included; OR > 1 reported more often in *validated ACEi angioedema cases*; OR < 1 reported more often in *validated ACEi controls*

^a^ in cases and controls, most data referring to the variable "time to onset" was incomplete or missing. The calculation of the completeness score is described in the Methods section: 2.2.3. **BfArM’s ADR-database**: documentation quality of validated cases.

^b^ *validated ACEi angioedema cases*: age unknown in 21 reports, gender unknown in 2 reports.

^c^ refers to current smoking at the time of the reported ADR. Former smokers were classified as non-smokers.

^d^ information about the amount of alcohol consumed (daily/weekly) was rare and may not have been reported. It was not possible to classify the cases in patients with a high or moderate alcohol consumption due to inaccurate information. Therefore, all cases in which any alcohol consumption was reported were counted, independent of the amount.

^e^ the term "allergy" refers to a reported allergy and the occurrence of any allergic and hypersensitivity reactions reported in the history of the patient.

^f^ the term "angioedema" summarizes previous angioedema or swellings coded in the SMQ "angioedema (narrow)" reported in the history of the patient.

^g^ refers to renal disorders, diabetes, asthma/COPD (chronic obstructive pulmonary disease) reported in the patients’ history or as a drug indication term for the used comedication.

^h^ the three ACEi monosubstances most frequently reported as "suspected/interacting" are tabulated. The remaining ACEi (not listed) were reported fewer than 5 times.

^i^ the analysis of the most frequently reported and most relevant comedications is based on monosubstances and combination products of the tabulated drug substances and/or drug classes and corresponds to the ATC classification. All drugs co-reported to the "suspected/interacting" ACEi were counted as "concomitant", regardless of whether they were reported as "suspected", "interacting" or "concomitant".

^j^ One ADR report may yield information about more than one seriousness criterion, therefore, the number of reported seriousness criteria exceeds that of the ADR reports.

Table 2 shows the absolute and relative number of reports and the calculated unadjusted and adjusted odds ratios for the reported demographic parameters, comorbidities, comedications, and seriousness criteria of *validated ACEi angioedema cases* and *controls* originating from Germany. Since there were 21 cases with missing data in the variables age and/or gender, multiple imputation methods were applied.

In contrast to the **EVDAS** analysis, more smokers (OR 4.8 [2.0–11.4]) and patients with concurrent calcium antagonist intake (OR 2.1 [1.1–4.0]) were among the *validated ACEi angioedema cases* compared to *validated ACEi controls* (unadjusted Odds Ratios, Table 2). However, only smoking (p-value: 0.043) remained statistically significantly after sensitivity analysis with multiple imputation. Concurrent intake of diuretics was reported statistically significantly more often in *validated ACEi controls* after logistic regression and multiple imputation (p-value: 0.023).

The reporting of smoking (OR 4.3 [1.8–9.9]) remained statistically significantly more often, and concurrent intake of diuretics (OR 0.4 [0.3–0.8]) remained reported statistically significantly less often in *matched validated ACEi angioedema cases* versus *ACEi controls (not validated)* after 1:2 matching by age and gender (S4 Table).

Furthermore, compared to the **EVDAS** analysis (i) "allergy" and "asthma" were not reported statistically significantly more frequently in the *validated ACEi angioedema cases*, (ii) "renal disorders" was not reported more frequently in *validated ACEi controls*, (iii) ramipril was much more frequently reported as "suspected/interacting" ACEi, in general.

### 2.) Comparative analysis of angioedema cases: ACEi versus ARBs and aliskiren

#### 2.1.) *EVDAS* analysis

Comparative analysis of angioedema cases between *ACEi* versus *ARBs* and *aliskiren* (each separately) revealed more females in *ARBs* (OR 0.7 [0.6–0.9]) and *aliskiren* cases (OR 0.6 [0.4–0.9]) than in *ACEi* cases (Table 1). In contrast, concurrent intake of acetylsalicyclic acid, analgesics, mTORi and fibrinolytics was more frequently reported in *ACEi* versus *ARBs* and *aliskiren angioedema cases*. A higher proportion of allergic patients was included in *ARBs* (6.8%) and *aliskiren* (13. 6%) *angioedema cases*, as well as patients with a history of urticaria in *ARBs angioedema cases* (0.9%) compared to *ACEi angioedema cases* (allergy: 4.3%, urticaria: 0.5%) (Table 1, S2 Table). *ACEi angioedema cases* were classified as "life-threatening" (15.5%) and led to or prolonged "hospitalization" (50.7%) the most frequently compared to the others.

### 2.2) *BfArM’s ADR-database* analysis

#### 2.2.1. Patient populations

Regarding the relevant information included in the calculation of the completeness score, the highest score was calculated for *ACEi angioedema cases* (0.74 [0.65–0.82]), followed by *ARBs* (0.67 [0.54–0.80]) and *aliskiren*, (0.68 [0.49–0.88]) *angioedema cases* (Table 2 and S5 Table).

In general, the proportion of allergic patients and patients with previous/recurrent angioedema in *validated ACEi* (12.4%, 24.0%), *ARBs* (19.0%, 11.1%) and *aliskiren angioedema* cases (24.2%, 35.4%) was much higher than in the **EVDAS** analysis (ACEi: 4.3% & 4.0%, ARBs: 6.8% & 4.5%, aliskiren: 13.6% & 4.9%) (Table 1, S2 Table). More patients with allergies were included in *validated ARBs* and *aliskiren angioedema cases* and more patients with a history of previous/recurrent angioedema in the *validated aliskiren angioedema cases* compared to the *validated ACEi angioedema cases*.

In eight (12.7%) of the *validated ARBs angioedema cases*, a history of prior ACEi therapy was reported. Reasons for discontinuing the previous ACEi therapy were "cough" (four times), "allergy" (once) and "angioedema" (once). In two cases, information was not available (NA).

In thirteen (39.4%) of the *validated aliskiren angioedema cases*, a history of prior ACEi and/or ARBs therapy was reported. As a reason for the discontinuation of the prior ACEi/ ARBs therapy, "angioedema" was reported seven times and "cough" twice. In four cases no information was available (NA).

#### 2.2.2. Number of ADR reports in relation to the number of drug prescriptions

The number of angioedema reports per 1,000 million drug prescriptions (in DDD) was higher for *ARBs* (10 angioedema reports) and *aliskiren* (154 angioedema reports) than for *ACEi* (8 angioedema reports) (Fig 2). Regarding the reported drug substances, the highest reporting rate (i.e. the number of ARD reports per 1,000 million drug prescriptions in DDD) compared to the other *ACEi/ ARBs* was found for lisinopril (13 angioedema reports) and valsartan (16 angioedema reports).

### 3.) Reported clinical phenotype

#### 3.1) *EVDAS* analysis

**Fig 3:**
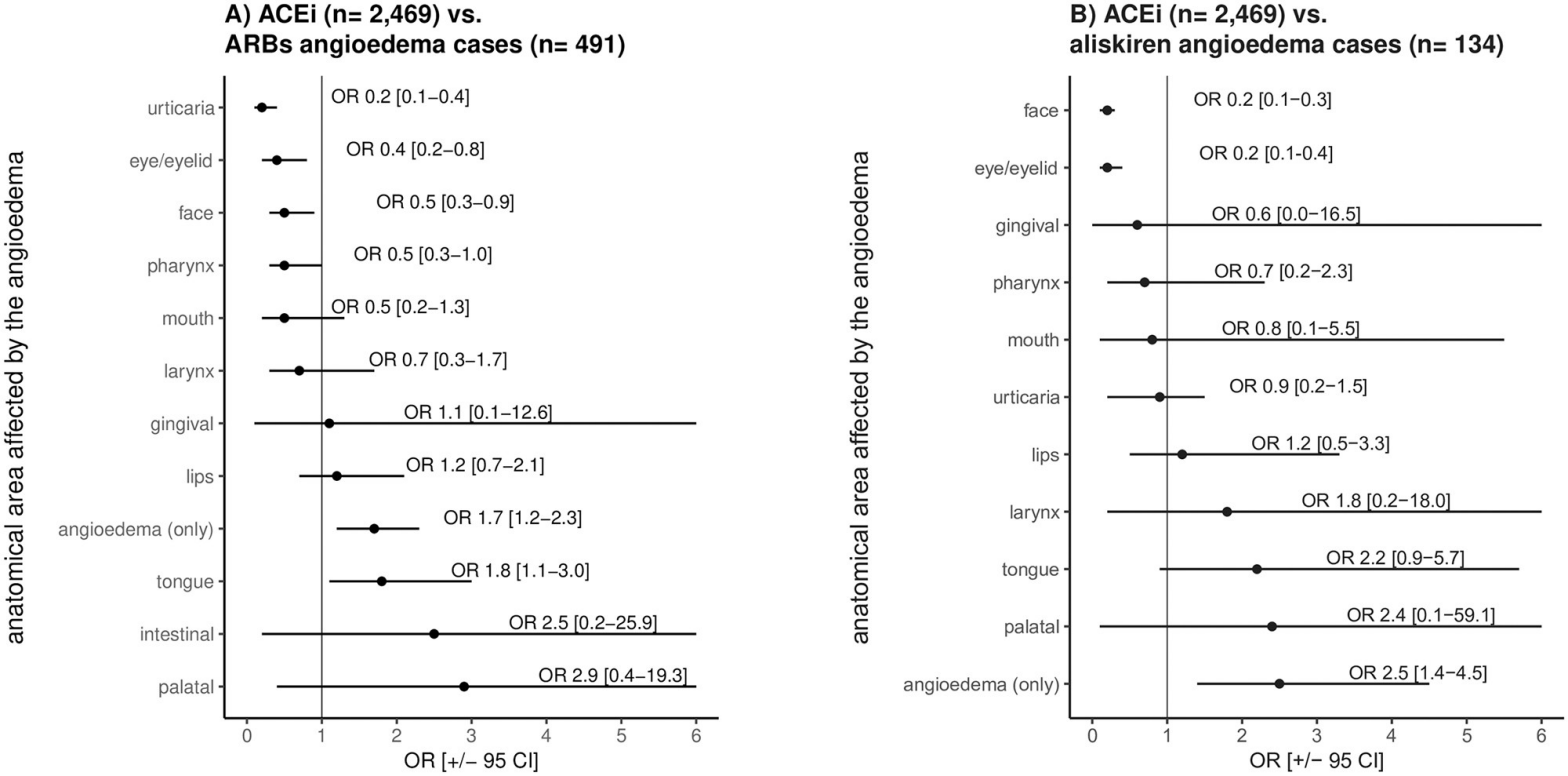
*EVDAS* analysis: Reported anatomical area affected by the angioedema according to SMQ "angioedema (narrow)" of the MedDRA terminology. *OR = 1 not included. OR > 1 more often reported in *ACEi angioedema cases*; OR < 1 more often reported in *ARBs* or *aliskiren angioedema cases*.

In *ACEi angioedema cases*, the "tongue" (19.4%) was mostly involved and more frequently reported in *ACEi* versus *ARBs* and *aliskiren angioedema cases* (Fig 3). In contrast, "face" and "eye/eyelid" were reported more frequently as affected anatomical areas in *ARBs* and *aliskiren angioedema cases* than in *ACEi angioedema cases*. In *ARBs* and *aliskiren angioedema cases*, "urticaria" (18.5%, 9.0%) and/or "pruritus" (9.2%, 13.4%) were reported more often as attendant symptoms than in *ACEi angioedema cases* ("urticaria": 5.0%, "pruritus": 3.1%). Additionally, "peripheral swellings/oedemas" were more frequently reported in *aliskiren* (23.1%) compared to *ACEi* (1.2%) and *ARBs* (2.6%) *angioedema cases*.

Fig 3 shows the calculated odds ratios with Bonferroni adjusted confidence intervals for the reported anatomical areas affected by the angioedema according to the SMQ "angioedema (narrow)" for *ACEi angioedema cases* versus *ARBs* and *aliskiren angioedema cases*. Therefore, only cases in which the respective RASi was reported as the "suspected" drug were included. For calculation of the odds ratios, the *ACEi angioedema cases* served as a reference. The number of ADR reports describing the same anatomical area e.g. "tongue oedema" and "swollen tongue" were merged into one group (here: tongue). In some of the reports, only the diagnosis "angioedema" was coded (designated as “only diagnosis angioedema”). One ADR report can contain more than one reported anatomical area affected by the angioedema. Therefore, the number of reported anatomical areas affected by the angioedema exceeds that of the ADR reports. Please note that some of the confidence intervals are not displayed completely.

In a stratified analysis of *ACEi angioedema cases* with concurrent use of mTORi (n = 42) or fibrinolytics (n = 38), the "tongue" was most often involved (31.0%, 31.6%) and more often involved than in the whole dataset (19.4%). Interestingly, none of these cases reported "urticaria" or "pruritus" (S7 Table).

#### 3.2) *BfArM’s ADR-database* analysis

In 15.7% of *validated ACEi angioedema cases*, only the summarized diagnosis "angioedema" was reported. As well as in **EVDAS**, the "tongue" was mostly involved in the *validated ACEi angioedema cases* (41.3%) (Table 3) in the **BfArM’s ADR-database**. In general, the proportion of reports yielding information about the anatomical area affected by the angioedema was much higher in **BfArM’s ADR-database** compared to the **EVDAS** analysis.

**Table 3:** *BfArM’s ADR-database* analysis: Stratified analysis of anatomical areas affected by ACEi-associated angioedemas.

	tongue ^a^	lips ^a^	face ^a^	pharynx ^a^	neck/throat ^a^	cheek ^a^	glottis ^a^	eye/eyelid ^a^
	41.3%	28.1%	20.6%	13.2%	12.4%	10.7%	9.1%	7.4%
	(n = 50)	(n = 34)	(n = 25)	(n = 16)	(n = 15)	(n = 13)	(n = 11)	(n = 9)
***patient demographics***								
mean age (median) [years]	66.0 (70)	64.9 (68.5)	65.7 (69)	66.6 (68.5)	66.1 (69)	65.4 (69)	65 (67)	57.8 (61)
female	46.0% (23)	32.4% (11)	64.0% (16)	31.3% (5)	40.0% (6)	30.8% (4)	36.4% (4)	77.8% (7)
male	54.0% (27)	67.6% (23)	36.0% (9)	68.8% (11)	60.0% (9)	69.2% (9)	63.6% (7)	22.2% (2)
***smoking habits*, *allergic conditions***								
smoker ^b^	26.0% (13)	5.9% (2)	12.0% (3)	12.5% (2)	13.3% (2)	7.7% (1)	36.4% (4)	11.1% (1)
allergy ^c^	8.0% (4)	20.6% (7)	16.0% (4)	6.3% (1)	13.3% (2)	7.7% (1)	0.0% (0)	33.3% (3)
angioedema ^d^	16.0% (32)	44.1% (15)	32.0% (8)	18.8% (3)	33.3% (5)	30.8% (4)	45.5% (5)	44.4% (4)
asthma/COPD ^e^	10.0% (5)	14.7% (5)	8.0% (2)	6.3% (1)	6.7% (1)	7.7% (1)	0.0% (0)	22.2% (2)
***comedication***								
everolimus	8.0% (4)	8.8% (3)	16.0% (4)	6.3% (1)	0.0% (0)	15.4% (2)	0.0% (0)	11.1% (1)
alteplase	2.0% (1)	0.0% (0)	0.0% (0)	0.0% (0)	0.0% (0)	0.0% (0)	0.0% (0)	0.0% (0)
***reported attendant reactions***								
urticaria ^f^	0.0% (0)	8.8% (3)	4.0% (1)	0.0% (0)	0.0 (0)	0.0 (0)	18.2% (1)	0.0 (0)
pruritus ^h^	0.0% (0)	14.7% (5)	16.0% (4)	0.0% (0)	6.7% (1)	7.7% (1)	0.0 (0)	33.3% (3)
***seriousness criteria*** ^i^								
serious	92.0% (46)	91.2% (31)	92.0% (23)	100.0% (16)	93.3% (14)	92.3% (12)	100.0% (11)	77.8% (7)
death	6.0% (3)	2.9% (1)	0.0% (0)	12.5% (2)	13.3% (2)	0.0% (0)	18.2% (1)	0.0% (0)
life-threatening	48.0% (24)	8.8% (3)	32.0% (8)	43.8% (7)	46.7% 7)	23.1 (3)	54.5% (6)	11.1% (1)
hospitalization	54.0% (27)	32.4% (11)	40.0% (10)	68.8% (11)	60.0% (9)	30.8% (4)	63.6% (7)	11.1% (1)

^a^ one report can yield information about more than one anatomical area affected by the angioedema. Therefore, the total number of areas affected by the angioedema exceeds that of the ADR reports.

^b^ refers to current smoking at the time of the reported ADR. Former smokers were classified as non-smokers.

^c^ the term "allergy" refers to a reported allergy and the occurrence of any allergic and hypersensitivity reactions reported in the history of the patient.

^d^ the term "angioedema" summarizes previous angioedema or swellings coded in the SMQ "angioedema (narrow)" reported in the history of the patient.

^e^ the term "asthma/COPD" refers to asthma/COPD (chronic obstructive pulmonary disease) reported in the patients’ history or as a drug indication for one of the drugs used concomitantly.

^f^ the term "urticaria" summarizes urticarias coded in the SMQ "angioedema (narrow)" reported as adverse drug reaction.

^h^ the term "pruritus" summarizes PTs that included pruritus independent of the anatomical area affected by the ADR.

^i^ one ADR report may yield information about more than one seriousness criterion. Thus, the number of reported seriousness criteria exceeds that of the ADR reports.

Table 3 shows the relative and absolute number of ADR reports of the stratified anatomical areas affected by ACEi-associated angioedemas. For each anatomical area affected by the angioedema, patient demographics, smoking habits and comorbidities, comedications, attendant symptoms and the seriousness criteria of the reports were analyzed. The information about anatomical areas affected by the angioedema was retrieved from the reported ADRs and the narratives of the angioedema reports.

With regard to the stratified analysis, patients in whom the "eye/eyelid" was involved were younger, more often females (77.8%) and the reports were less often designated as "serious". Additionally, "allergy" and "pruritus" as attendant symptoms were mentioned in one third of these reports. Patients in whom the "cheek", "pharynx", "glottis" or "neck/throat" were affected were more often males and the reaction was described more often as "serious". "Urticaria" and "pruritus" did not occur in patients in whom the "tongue" and the "pharynx" were involved.

A higher proportion of "face" and "eye/eyelid" involvement was also observed in *validated ARBs* (34.9%, 12.7%) and *aliskiren* (39.4%, 12.1%) *angioedema cases* versus va*lidated ACEi angioedema cases* (20.6%, 7.4%) (Table 3 and S5 Table). The same applies for "pruritus" and "urticaria" (*validated ARBs angioedema cases*: 15.9%, 12.7%, *validated aliskiren angioedema cases*: 15.2%, 9.1%, *validated ACEi angioedema cases*: 5.0%, 3.3%).

### 4.) *BfArM’s ADR-database* analysis: Time-to-onset of angioedema reactions

**Fig 4:**
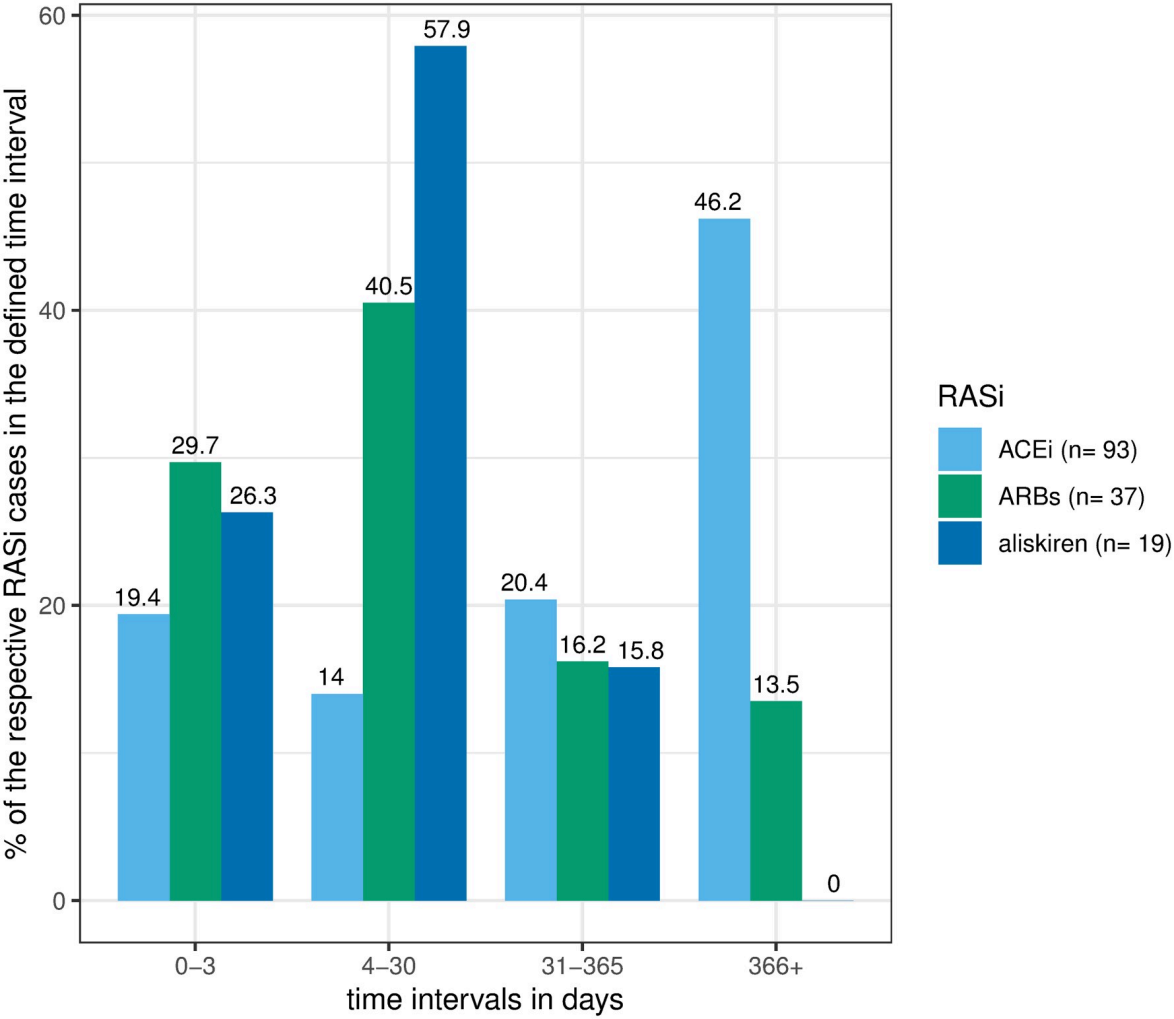
BfArM’s ADR-database analysis: "Time-to-onset" analysis of the angioedema reaction. Fig 4 shows the "time-to-onset" analysis of validated ACEi, ARBs, and aliskiren-associated angioedemas. In this figure only cases providing information on the "time-to-onset" were included.

In 76.9% of *validated ACEi*, 58.7% of *validated ARBs*, and 57.6% of *validated aliskiren angioedema cases* data on the "time-to-onset" variable was available (Fig 4). Compared to *ACEi* (33.3%) a higher proportion of *validated ARBs* (70.3%) and *aliskiren* (84.2%) *angioedema cases* reported that the angioedema occurred during the first month of therapy. In contrast, the reactions occurred after the first year in a much higher proportion in *validated ACEi angioedema cases* 46.2% compared to ARBs (13.5%) and aliskiren (0.0%).

### 5.) *BfArM’s ADR-database* analysis: Treatment of angioedema

Information about the treatment of angioedemas was available in 64.4% of the *validated ACEi angioedema cases* (S8 Table). Most of the patients were treated with antihistamines and/or steroids, only (60.3%, n = 47). Of the patients treated with antihistamines and/or steroids, 15 had a rapid and 13 a slow regression of symptoms (19 cases: not assessable). Circulation stabilizing drugs (12.8%, n = 10) were used additionally to antihistamines and/or steroids (n = 9) or alone (n = 1) and led to a rapid regression in two patients and a slow regression in seven patients (in one patient not assessable). A medical intervention (e.g. intubation) was performed in 16.7% (n = 13) of the cases. C1-esterase inhibitors were used in 10.3% (n = 8) of the cases (n = 6 rapid regression, n = 1 slow regression, n = 1 unknown). Icatibant was administered in 5.1% of the cases and in one case additional fresh frozen plasma was administered. Both treatments led to a rapid regression of symptoms in all patients.

In 79.3% of the *validated ACEi angioedema cases*, information about "action taken with regard to the administered ACEi" was available. "Drug withdrawn” was reported in 92.7% of these cases.

Treatment was only rarely reported for ARBs (30.2% of cases) and aliskiren (18.2% of cases)-associated angioedemas. In those cases in which information about angioedema treatment was available, antihistamines and/or steroids were used. "Drug withdrawn" was reported in 86.7% (39/45) of *ARBs*, and 96.0% (24/25) of *aliskiren angioedema cases* (related to the number of reports that included information about action taken with regard to the administered drug).

## Discussion

To the best of our knowledge, the present study represents the first retrospective analysis of angioedema reports associated with RASi covering the entire EEA performed in **EVDAS**. To strengthen the significance of this analysis, an additional analysis of validated cases originating from Germany was performed in **BfArM’s ADR-database**.

Many studies have been published in which associated factors of ACEi-induced angioedemas were analyzed. However, only a few investigated associated factors of ARBs and aliskiren-associated angioedemas. In our analysis, already known associations of ACEi angioedemas were found and some of them were also observed for ARBs and aliskiren angioedemas (e.g. "previous/recurrent angioedema"). Differences were noted between ACEi, ARBs and aliskiren with regard to the reported seriousness criteria (ACEi-associated angioedemas were more "serious"), the reporting rates (higher rates for ARBs and aliskiren) and the clinical phenotypes ("urticaria" reported more often for ARBs and aliskiren-associated angioedemas). The analysis performed in **EVDAS** and **BfArM’s ADR-database** showed similarities (e.g. clinical phenotypes) but also differences (e.g. smoking habits).

### Patient demographics and gender-stratified analysis

Female gender has been reported by other authors [1, 5, 19, 53] as a risk factor for developing an ACEi-associated angioedema. In our analysis, ACEi-associated angioedemas occurred 1.5 times more often in females than in males when the *validated ACEi angioedema cases* were put in relation to the number of assumed ACEi-exposed patients [33]. Regardless of any patient-related exposure data (which were not available for aliskiren in DEGS1), more females were included in *aliskiren angioedema cases* compared to their controls. In order to make conclusive statements, gender-related drug exposure with aliskiren has to be considered.

Gender-stratified analysis showed that the association with smoking was more pronounced in males than in females whereas it was the opposite regarding allergic conditions. This finding possibly reflects gender-specific diseases or behaviors [54, 55]. A previous German health study diagnosed more females as being allergic (35.8%) and/or asthmatic (9.9%) than males (24.1% allergic, 7.3% asthmatic), while surveys investigating smoking behavior reported more male than female smokers [54, 55].

### Allergic conditions, comorbidities and reported seriousness criteria

A pre-existing history of urticaria and angioedema, as well as allergic and asthmatic conditions were reported more often in all of the three *RASi angioedema cases* compared to their *controls*. Seasonal allergies [19] and previous angioedemas [11] are also described as associated factors in ACEi angioedemas in literature.

In all three controls of the **EVDAS** analysis, more patients with a history of renal disorders were involved compared to their respective cases. This was not observed in the analysis of validated **BfArM** cases. However, this finding in the **EVDAS** analysis most likely reflects an association with the ADRs reported in the controls (e.g. acute kidney injury) and should therefore not be interpreted as a protective factor for RASi-associated angioedema [19]. It has to be noted that our analysis of renal disorders did not differentiate between acute and chronic kidney disease, which are substantially different clinical entities. This was the case because a proper assignment to one of the used SMQs was not possible since both SMQs have some preferred terms in common resulting in an overlap [45].

ACEi-associated angioedemas were most often designated as "life-threatening" and most often led to or prolonged "hospitalization" compared to ARBs and aliskiren. In this regard, Toh et al. [8] also discussed a more serious course of ACEi-associated angioedemas compared to ARBs and aliskiren.

### Reported smoking habits, comedications and clinical phenotypes in relation to the assumed pathophysiological mechanism of interaction

With regard to pathophysiology, one can roughly distinguish between histamine-mediated and bradykinin-mediated angioedemas [2, 12]. The following section offers a brief discussion of the differences between the clinical phenotypes of histamine-mediated and bradykinin-mediated angioedemas and the mechanism of interaction with some comedications in relation to the results of our analysis.

Both histamine and bradykinin can induce vasodilatation and increased vascular permeability leading to angioedema [12]. Histamine is either released from mast cells and/or basophils in context with an allergic, immunoglobuline E (IgE) mediated reaction or via non-immunological mechanisms [56]. Histamine-mediated reactions typically present with urticaria and pruritus [2, 12, 57] and respond to antihistamines [57, 58]. Bradykinin-mediated angioedema result from an interference in or inbalance of the bradykinin degradation pathway [2, 11, 12]. This may occur due to a hereditary defect or through external factors (e.g. ACEi). In contrast to histamine-mediated angioedemas, bradykinin-mediated angioedemas dot not usually present with "urticaria" and "pruritus".

When ACE is blocked, e.g. by ACEi, bradykinin can be degraded by alternative enzymes such as DPPIV and/or neutral endopeptidase (NEP). A decreased level of DPPIV activity was measured in patients during ACEi-associated angioedema attacks [59]. Therefore, drugs and/or comorbidities that have an impact on bradykinin levels by blocking or reducing DPPIV activity may influence the occurrence of angioedemas [59, 60].

Smoking has been described as a risk factor for ACEi-associated angioedemas [21, 59] and is assumed to lead to a reduced DPPIV activity [13, 60]. In our analysis, this was only observed for the comparison of *validated ACEi angioedema cases* with *validated ACEi controls* (p-value: 0.043) (**BfArM’s ADR-database)**. An underreporting of smoking habits in **EVDAS** may be one possible explanation that this finding was not observed in **EVDAS**.

In our analysis, exposure to fibrinolytics (e.g. tissue plasminogen activators (tPA)) was 16.5 fold higher in *ACEi angioedema cases* versus their *controls*. Angioedema is described to occur in 1.7 [61] -7.9% [62] of all cerebral vascular accident patients treated with tissue plasminogen activators (tPA) and is reported to occur more frequently when ACEi is taken concomitantly [61, 62, 63]. The increased risk of angioedema may result from neuronal damages leading to an upregulation of bradykinin-receptors-B2, and/or the increased production of bradykinin induced by tPA [62, 63].

mTORi therapy was reported about 9.2 times more often in the *ACEi angioedema cases* versus their *controls* and about 4.3 times more often versus *ARBs angioedema cases*. A greater number of angioedema events per 100 treatment years was estimated in kidney transplant patients treated with mTORi with combined ACEi therapy (3.8) than with combined ARBs therapy (0.5) [64]. The DPPIV activity in patients with renal transplants is generally expected to be lower [61, 65]. Additionally, the DPPIV activity in cultured endothelial cells was decreased by up to 60.0% when treated with sirolimus [65].

However, in our analysis the number of cases with concurrent mTORi and fibrinolytics use in *ARBs* and *aliskiren angioedema cases* was rather low or no cases were available. Either, those drugs potentiate the angioedema risk only when combined with ACEi, or the combined therapies with ARBs and aliskiren are too seldom to be observed in our analysis.

Interestingly, in none of the *ACEi angioedema cases* with concurrent fibrinolytics or mTORi therapy "urticaria" and/or "pruritus" was mentioned. Hence, a bradykinin-mediated angioedema appears plausible as also described in literature [62, 63, 64, 65]. For both, the "tongue" was the anatomical area most often affected by the angioedema. In the stratified analysis, none of the validated cases in which the "tongue" was involved presented with "urticaria" or "pruritus" as attendant symptoms. Hence, based on our observations one may speculate whether involvement of the "tongue" could be more often associated with bradykinin-mediated angioedemas.

With regard to diabetes, some studies reported that ACEi-associated angioedemas occurred less frequently in patients with diabetes [5, 19, 65]. Byrd et al. reported a less frequent occurrence of ACEi- and NEP-associated angioedemas in ACE treated patients with diabetes and measured a higher DPPIV actitvity in ACEi treated diabetic patients compared to ACEi treated non-diabetic patients [13]. In line with these findings, in our analysis the proportion of patients taking any antidiabetic drugs (interpreted as patients with diabetes) excluding DPPIV inhibitors was slightly lower in all RASi angioedema cases versus RASi controls in the **EVDAS** analysis. However, patients concomitantly treated with DPPIV inhibitors may have an increased risk of developing ACEi-associated angioedemas potentiated by the inhibition of the enzyme DPPIV [11, 66]. In our analysis, a higher DPPIV inhibitor use compared to the respective controls was observed in *ARBs* and *aliskiren angioedema cases* only. In literature, conflicting data exists whether the combined therapy of DPPIV inhibitors and ARBs may potentiate the occurrence of angioedemas [66, 67].

Concerning the anatomical areas affected by the angioedema, a higher proportion of "face" and/or "eye/eyelid" involvement was reported in *ARBs* and *aliskiren angioedema cases* compared to *ACEi angioedema cases*. The same applies to "urticaria" and "pruritus", as well as to "peripheral swellings/oedemas". Others reported that angioedemas that involved the "eye" are significantly more often histamine-mediated angioedemas [53] while peripheral swellings are more frequently observed in bradykinin-mediated angioedemas [53, 57]. Likewise, the proportion of reported "allergy" in the patients’ history and "pruritus" as an ADR was highest in the stratified analysis of the validated cases in which the "eye/eyelid" was involved (small sample size: n = 9). Slightly more "peripheral swellings" caused by aliskiren vs. ARBs and ACEi were also described in literature [24]. However, "peripheral swellings/oedemas" may also be a symptom of target diseases for which the ACEi or ARB is taken (e.g. heart failure).

It should be noted, though, that inaccuracies in reporting like the use of "face" as an umbrella term, or reporting only the diagnoses "angioedema" might have impacted the results. However, the results from **EVDAS** were confirmed in our full-text analysis of the *validated RASi angioedema cases* (**BfArM’s ADR-database**).

Antihistamines and glucocorticoids are used as standard therapy to treat angioedemas in German emergency departments [58]. Theoretically, both should only be effective in histamine-mediated angioedemas [10]. C1-inhbitors, fresh frozen plasma and icatibant are not approved for the treatment of drug-induced angioedemas. They may be used off-label and should lead to a clinical response in hereditary and bradykinin-mediated angioedemas. In our analysis of validated angioedema cases, C1 inhibitors, fresh frozen plasma and icatibant were only used to treat ACEi-associated angioedemas. Antihistamines and glucocorticoids were most frequently used to treat ACEi-associated angioedemas, but did not lead to any improvement in almost half of the *validated ACEi angioedema cases* (where information regarding the clinical response was available). However, a clear allocation of whether the angioedema was histamine-mediated or bradykinin-mediated is still not possible based on the treatment success of the applied therapy, since angioedema can also regress spontaneously [56]. Medical interventions (e.g. intubations) were only reported in ACEi-associated angioedemas. This reflects the more serious course of ACEi-associated angioedemas in our cases [8]. In general, information about the treatment of ARBs and aliskiren-associated angioedemas was rare, and if available, showed that antihistamines and/or glucocorticoids were used.

ARBs and aliskiren-associated angioedemas occurred more often within the first month of therapy, whereas 46.2% of ACEi-associated angioedemas occurred even after one year of therapy. The occurrence of ACEi-associated angioedemas after several years of ACEi therapy is known [16, 68]. In contrast, this is not described in literature for ARBs and aliskiren-associated angioedemas.

In summary, one may speculate that there is a higher proportion of histamine-mediated angioedemas in *ARBs* and *aliskiren angioedema cases*, based on the observed differences of clinical phenotypes, treatment response and "time-to-onset" of angioedema reactions. However, this cannot be concluded with certainty based on our results, since (among others) laboratory investigations are lacking. The differences observed could also have been influenced by differences between the involved patient populations, e.g. more patients with allergies, and/or previous/recurrent angioedemas as well as ADRs, with previous drug therapies being included in *ARBs* and *aliskiren angioedema cases*.

### *BfArM’s ADR-database* analysis: Administered drug classes and drug substances in relation to the number of drug prescriptions

As described above, angioedema incidences associated with ACEi use are reported to be higher than that of ARBs [8]. Regarding aliskiren-associated angioedemas, conflicting incidences have been published [8, 23, 24].

In our analysis, the largest number of angioedema reports in relation to the number of drug prescriptions in 1,000 million DDD was calculated for aliskiren (154 reports) followed by ARBs (10 reports) and ACEi (8 reports). However, it is possible that ACEi-associated angioedemas may be reported less frequently than those associated with ARBs and aliskiren, since physicians tend to report known or expected ADRs less [69]. In contrast, unexpected ADRs (potentially aliskiren, ARBs-associated angioedemas) as well as ADRs associated with novel drug therapies are more likely to be reported [69]. With regard to the proportion of angioedema reports in relation to all ADR reports, we observed the highest proportion for ACEi (20.3%) and the lowest (7.6%) for ARBs (S6 Table).

Regardless of any exposure data, ramipril (67.8%) was the ACEi and valsartan (33.3%) the ARB most often reported. This finding is in line with ramipril being the ACEi with the largest exposure in Germany [32]. However, in the context of the number of drug prescriptions, slightly more angioedema reports were calculated for lisinopril (13 reports) and enalapril (9 reports) than for ramipril (7 reports). A higher angioedema incidence for lisinopril or enalapril has not previously been described. However, these marginal differences may more likely be coincidental.

In the **EVDAS** analysis, losartan was the ARB reported the most in *ARBs angioedema cases* and more often reported in *ARBs angioedema cases* versus *controls* (OR 1.7 [1.4–2.1]). This is in line with data reported in literature. Toh et al. suspected a higher angioedema incidence for losartan than for other ARBs with an incidence of 2.28 (1.84–2.79) per 1,000 person-years [8]. However, losartan ranked only third in **BfArM’s ADR-database** analysis, and in relation to the number of drug prescriptions, higher reporting rates were calculated for irbesartan (18 reports), valsartan (16 reports) and telmisartan (13 reports).

### *EVDAS* analysis versus *BfArM’s ADR-database* analysis

Some of the analyses undertaken in both databases yielded the same results. However, as in the analysis of clinical phenotypes, the proportion of cases in the subgroups mostly increased in the full-text analysis performed in **BfArM’s ADR-database**. Ramipril and smoking were reported more often in *validated ACEi angioedema cases* of **BfArM’s ADR-database** compared to the **EVDAS** analysis. Differences in prescribing behavior (e.g., ramipril being the ACEi most frequently prescribed in Germany) and reporting behavior regarding life-style factors such as smoking among the EEA countries may account for these discrepancies. In summary, the high-level analysis seems to be sufficient to predict the direction of the results.

### Strengths and limitations of the analysis

The major strengths of this analysis are the huge number of ADR reports collected over a long period of time in a diverse study population, as well as the case validation, which mainly supports the results of the high-level evaluation performed in EVDAS. One limitation is the lack of matching exposure data. Data from the German drug prescription reports are not patient-related and represent the number of drug prescriptions in defined daily doses only, which may differ from the actually prescribed and/or administered dose [32]. Additionally, not all ADRs that occur are reported and the proportion of this underreporting [70] is unknown. Additionally, the underreporting may differ depending on the drug administered and the nature of the ADR experienced. As a consequence of these both limitations, exact incidences and prevalences cannot be calculated, which also applies to our results. To address this limitation, we set the number of ACEi reports in relation to the number of assumed drug-exposed inhabitants. This allows for an estimation of the dimension but should not be misunderstood as exact prevalences and/or incidences. Unfortunately, patient-related data about ARBs and aliskiren use in the German population was not available [33], Therefore the calculation could not be carried out for ARBs and aliskiren. Furthermore, the quality of the analysis depends on the information provided in each ADR report and may differ between patient populations and countries.

## Conclusion

Some of the risk factors already known for ACEi angioedemas were confirmed in our analysis and were also seen in ARBs and aliskiren-associated angioedemas. Differences between ACEi vs. ARBs and aliskiren regarding the reported clinical phenotypes, the "time-to-onset" and the treatment of angioedemas and their response to the treatment, but also between the patient populations involved were observed. However, it needs to be clarified if the observed differences reflect different pathophysiologies or if differences between the patient populations involved may account for these findings. Due to the limitations of analysis in spontaneous report databases, further research, is needed to complement our data.

## Supporting information

S1 File *EVDAS* analysis: Sacubitril/valsartan-associated angioedemas. (DOCX)

S1 Table *EVDAS* analysis: Gender-stratified analysis of reported characteristics in *ACEi angioedema cases*. * OR = 1 is not included; OR > 1 reported more often in females; OR < 1 reported more often in males. ^a^ age unknown: *ACEi angioedema cases*: 179 cases (5.4% of cases), *ACEi controls*: 717 cases (6.7% of cases). ^b^ refers to current smoking at the time of the reported ADR. Former smokers were classified as non-smokers. ^c^ the term "allergy" refers to a reported allergy and the occurrence of any allergic and hypersensitivity reactions reported in the history of the patient. ^d^ skin and subcutaneous tissue disorders were analyzed based on the SOC "skin and subcutaneous tissue disorders", urticaria based on the HLT "urticarias". The term "angioedema" summarizes previous angioedema or swellings coded in the SMQ "angioedema (narrow)" reported in the history of the patient. ^e^ suitable hierarchical levels of the MedDRA terminology were chosen for analysis of the reported patients’ comorbidities. The term "renal disorders" was identified using the SMQs "acute renal failure" and "chronic kidney disease"; "diabetes": SMQ "hyperglycaemia/new onset diabetes mellitus"; "asthma": SMQ "asthma/bronchospasm"; "malignant tumors": SMQ "malignant tumours"; "thyroid disorders": SMQ "thyroid dysfunction". ^f^ the four ACEi monosubstances most frequently reported as "suspected/interacting" are tabulated. The relative number of ADR reports specifying one of the other ACEi (not listed) as "suspected/interacting" was lower than 2%. One ADR report may contain more than one ACEi as "suspected/interacting" drug substance. Thus, the number of reported ACEi exceeds that of the ADR reports. ^g^ the analysis of the most frequently reported and most relevant comedications is based on monosubstances and combination products of the tabulated drug substances and/or drug classes and corresponds to the ATC classification. All drugs co-reported to the "suspected/interacting" ACEi were counted as concomitant, regardless of whether they were reported as "suspected", "interacting" or "concomitant". ^h^ deviating from the ATC-code, the analysis concerning "analgesics" also includes ADR reports in which ibuprofen and/or diclofenac were listed as suspected/interacting or concomitant drug. We excluded ADR reports in which acetylsalicyclic acid was listed as suspected/interacting or concomitant drug. The number of ADR reports in which acetylsalicyclic acid was used concurrently were analyzed separately. ^i^ deviating from the ATC-code, we excluded ADR reports in which a DPPIVi was listed as suspected/interacting or concomitant drug in the analysis concerning "diabetics". The number of ADR reports in which DPPIVi was used concurrently was analyzed separately. ^j^ one ADR report may yield information about more than one seriousness criterion. Thus, the number of reported seriousness criteria exceeds that of the ADR reports. S1 Table shows the absolute and relative number of reports and the calculated unadjusted and adjusted odds ratios of females versus males for the reported demographic parameters, comorbidities, comedications and seriousness criteria of the gender-stratified *ACEi angioedema cases* of the European Economic Area (EEA).
(PDF)

S2 Table *EVDAS* analysis: Reported characteristics in *ARBs* and *aliskiren angioedema cases* and *ARBs* and *aliskiren controls*. *OR = 1 is not included; OR > 1 reported more often in *ARBs* or *alsikiren angioedema cases*; OR < 1 reported more often in *ARBs* or *aliskiren controls*. ^a^ age unknown: *ARBs angioedema cases*: 88 cases (12.8% of cases), *ARBs controls*: 1,276 cases (14.9% of cases), *aliskiren angioedema cases*: 37 cases (22.8% of cases), *aliskiren controls*: 242 cases (23.8% of cases). ^b^ only current smoking at the time of the reported ADR was counted. Former smokers were classified as non-smokers. ^C^ the term "allergy" summarizes allergic and hypersensitivity reactions reported in the history of the patient. ^d^ skin and subcutaneous tissue disorders were analyzed based on the SOC "skin and subcutaneous tissue disorders", urticaria based on the HLT "urticarias". The term "angioedema" summarizes previous angioedema or swellings coded in the SMQ "angioedema (narrow)" reported in the history of the patient. ^e^ suitable hierarchical levels of the MedDRA terminology were chosen for the analysis of the reported patients’ comorbidities. The term "renal disorders" was identified using the SMQs "acute renal failure" and "chronic kidney disease"; "diabetes": SMQ "hyperglycaemia/new onset diabetes mellitus"; "asthma": SMQ "asthma/bronchospasm"; "malignant tumors": SMQ "malignant tumours"; "thyroid disorders": SMQ "thyroid dysfunction". ^f^ the three ARB monosubstances most frequently reported as "suspected/interacting" are tabulated. One ADR report may contain more than one ARB as "suspected/interacting" drug substance. Thus, the number of reported ARBs exceeds that of the ADR reports. ^g^ the analysis of the most frequently reported and most relevant comedications is based on monosubstances and combination products of the tabulated drug substances and/or drug classes and corresponds to the ATC classification. All drugs co-reported to the "suspected/interacting" ARBs were counted as concomitant, regardless of whether they were reported as "suspected", "interacting" or "concomitant". ^h^ deviating from the ATC-code, the analysis concerning "analgesics" also includes ADR reports in which ibuprofen and/or diclofenac were listed as suspected/interacting or concomitant drug. We excluded ADR reports in which acetylsalicyclic acid was listed as suspected/interacting or concomitant drug. The number of ADR reports in which acetylsalicyclic acid was used concurrently were analyzed separately. ^i^ deviating from the ATC-code, we excluded ADR reports in which a DPPIVi was listed as suspected/interacting or concomitant drug in the analysis concerning "diabetics". The number of ADR reports in which DPPIVi was used concurrently was analyzed separately. ^j^ one ADR report may yield information about more than one seriousness criterion, therefore, the number of reported seriousness criteria exceeds that of the ADR reports. S2 Table shows the absolute and relative number of reports and the calculated unadjusted and adjusted odds ratios for the reported demographic parameters, comorbidities, comedications and seriousness criteria of *ARBs* and *alsikiren angioedema cases* versus their respective *controls* of the European Economic Area (EEA).
(PDF)

S3 Table Mean number of angioedema and ADR reports (total) in relation to the number of assumed ACEi-exposed inhabitants/males/females. ^a^ the calculation of the mean number of angioedema reports, controls (ADR reports without angioedema reports) and ADR reports (total) per 1 million ACEi-exposed inhabitants/males/females was restricted to the years 2010–2016. This was the case since the ADR reports were analyzed for only half of the year 2017 (analysis criteria 01/01/2010-30/06/2017). S3 Table shows the calculated mean number of angioedema and ADR reports in relation to the assumed number of ACEi-exposed inhabitants/males/females per 1 million assumed ACEI-exposed inhabitants/males/females in Germany. The number of inhabitants per year was extracted from the GENESIS database [50] and multiplied by the proportional share of ACEi exposure in the German population published in DEGS1 [33]. A proportion of about 17.5% of German adults, 19.0% of German adult males, and 16.0% of German adult females taking an ACEi were extracted from the published graphic in DEGS1.
(PDF)

S4 Table *BfArM’s ADR-database* analysis: Characteristics of *matched validated ACEi angioedema cases* and *ACEi controls (not validated)*. *OR = 1 is not included; OR > 1 reported more often in *matched validated ACEi angioedema cases*; OR < 1 reported more often in *matched ACEi controls (not validated)* ^a^ in 7 of the *validated ACEi angioedema cases* neither age or gender (or both) were reported, hence 114 cases remained. The 1:2 matching by age and gender to the *ACEi controls (not validated)* was only performed for the cases in which age and gender were reported. ^b^ refers to current smoking at the time of the reported ADR. Former smokers were classified as non-smokers. ^c^ the term "allergy" refers to a reported allergy and the occurrence of any allergic and hypersensitivity reactions reported in the history of the patient. ^d^ the term "angioedema" summarizes previous angioedema, or swellings coded in the SMQ "angioedema (narrow)" reported in the history of the patient. ^e^ refers to the respective comorbidity reported in the patients’ history or as a drug indication tem for the used comedication. ^f^ the analysis of the most reported and most relevant comedications is based on monosubstances and combination products of the tabulated drug substances and/or drug classes and corresponds to the ATC classification. All drugs co-reported to the "suspected/interacting" ACEi were counted as concomitant, irrespective if they were reported as "suspected", "interacting", or "concomitant". ^g^ one ADR report may inform about more than one seriousness criterion. Thus, the number of reported seriousness criteria exceeds the number of ADR reports. S4 Table shows the absolute and relative number of reports and the calculated unadjusted odds ratios for the reported demographic parameters, comorbidities, comedications, and seriousness criteria of the *matched validated ACEi angioedema cases* and *matched ACEi controls (not validated)*.
(PDF)

S5 Table *BfArM’s ADR-database* analysis: Characteristics of *validated ARBs* and *aliskiren angioedema cases*. ^a^ age unknown: *validated ARBs angioedema cases*: 21 cases (33.3% of cases), *validated aliskiren angioedema cases*: 13 cases (39.4% of cases). ^b^ refers to current smoking at the time of the reported ADR. Former smokers were classified as non-smokers. ^c^ the term "allergy" refers to a reported allergy and the occurrence of any allergic and hypersensitivity reactions reported in the history of the patient. ^d^ the term "angioedema" summarizes previous angioedema, or swellings coded in the SMQ "angioedema (narrow)" reported in the history of the patient. ^e^ refers to the respective comorbidity reported in the patients’ history or as a drug indication tem for the used comedication. ^f^ the analysis of the most reported and most relevant comedications is based on monosubstances and combination products of the tabulated drug substances and/or drug classes and corresponds to the ATC classification. All drugs co-reported to the respective "suspected/interacting" drug substance were counted as concomitant, irrespective if they were reported as "suspected", "interacting", or "concomitant". ^g^ one ADR report may inform about more than one seriousness criterion. Thus, the number of reported seriousness criteria exceeds the number of ADR reports. ^h^ one ADR report may inform about more than one anatomical area affected of the angioedema. Thus, the number of reported anatomical areas affected of the angioedema exceeds the number of ADR reports. ^i^ one ADR report may inform about more than one attendant symptom. Thus, the number of reported attendant symptoms exceeds the number of ADR reports. S5 Table shows the absolute and relative number of the reported characteristics of the *validated ARBs* and *aliskiren angioedema cases*.
(PDF)

S6 Table Number of *ACEi*, *ARBs*, *and aliskiren angioedema cases* and their total number of ADR reports in relation to the number of drug prescriptions in Germany (2010–2016). ^a^ all identified cases (not validated) in **BfArM’s ADR-database** analysis of the time period 01/2010-12/2016. ^b^ cumulative number of drug prescriptions (monosubstances) for the years 2010–2016 [34]. ^c^ all angioedema reports including reports from 2017. The administered reported dose was analyzed during the validation process based on the complete report (including narratives; see Material and methods). ^d^ definition of ATC-code and the respective DDD of ACEi, ARBs and aliskiren monosubstances [41, 42]. ^e^ the incidences were taken from a meta-analysis of randomized trials performed by Makani et al. [23]. ^f^ number of ACEi reports with concomitant use of everolimus. ^g^ number of drug prescriptions for everolimus [34]. S6 Table shows the absolute and relative number of *ACEi*, *ARBs and aliskiren angioedema cases* and their total number of ADR reports in the time periode 01/2010-12/2016 as well as their relation to the number of drug prescriptions in 1,000 Mio DDD. Additionally, the number of angioedema reports per drug prescriptions fitted to the administered dose versus defined daily dose (DDD) ratio was calculated.
(PDF)

S7 Table *EVDAS* analysis: Reported characteristics in *ACEi angioedema cases* with concurrent mTORi, fibrinolytics, or DPPIVi use. ^a^ age unknown: *ACEi angioedema cases* with concomitant mTORi therapy: 3 cases (7.1% of cases), *ACEi angioedema cases* with concomitant fibrinolytics therapy: 2 cases (5.3% of cases), *ACEi angioedema cases* with concomitant DPPIVi therapy: 6 cases (9.0% of cases). ^b^ current smoking at the time of the reported ADR was count, only. Former smokers were classified as non-smokers. ^c^ the term "allergy" summarizes allergic and hypersensitivity reactions reported in the history of the patient. ^d^ skin and subcutaneous tissue disorders were analyzed based on the SOC "skin and subcutaneous tissue disorders", urticaria based on the HLT "urticarias". The term "angioedema" summarizes previous angioedema, or swellings coded in the SMQ "angioedema (narrow)" reported in the history of the patient. ^e^ suitable hierarchical levels of the MedDRA terminology were chosen for the analysis of the reported patients’ comorbidities. The term "renal disorders" was identified using the SMQs "acute renal failure" and "chronic kidney disease"; "diabetes": SMQ "hyperglycaemia/new onset diabetes mellitus"; "asthma": SMQ "asthma/bronchospasm"; "malignant tumors": SMQ "malignant tumours"; "thyroid disorders": SMQ "thyroid dysfunction". ^f^ tabulated are the four ACEi monosubstances reported as "suspected/interacting" most frequently (of all cases). One ADR report may contain more than one ACEi as "suspected/interacting" drug substance. Thus, the number of reported ACEi exceeds the number of ADR reports. ^g^ the analysis of the most frequently reported and most relevant comedications is based on monosubstances and combination products of the tabulated drug substances and/or drug classes and corresponds to the ATC classification. All drugs co-reported in *ACEi angioedema cases* with concurrent mTORi, fibrinolytics or DPPIVi use were counted as concomitant, regardless of whether they were reported as "suspected", "interacting" or "concomitant". ^h^ deviating from the ATC-code, the analysis concerning "analgesics" also includes ADR reports in which ibuprofen and/or diclofenac were listed as suspected/interacting or concomitant drug. We excluded ADR reports in which acetylsalicyclic acid was listed as suspected/interacting or concomitant drug. The number of ADR reports in which acetylsalicyclic acid was used concurrently were analyzed separately. ^i^ deviating from the ATC-code, we excluded ADR reports in which a DPPIVi was listed as suspected/interacting or concomitant drug in the analysis concerning "diabetics". The number of ADR reports in which DPPIVi was used concurrently was analyzed separately. ^j^ one ADR report may yield information about more than one seriousness criterion. Thus, the number of reported seriousness criteria exceeds that of the ADR reports. ^k^ one ADR report may yield information about more than one anatomical area affected of the angioedema. Thus, the number of reported anatomical areas affected of the angioedema exceeds that of the ADR reports. ^l^ one ADR report may yield information about more than one attendant symptom. Thus, the number of reported attendant symptoms exceeds that of the ADR reports. S7 Table shows the absolute and relative number of reports for the reported demographic parameters, comorbidities, comedications, and seriousness criteria, anatomical areas affected by the angioedema, and attendant symptoms of *ACEi angioedema cases* with concurrent mTORi, fibrinolytics or DPPIVi use of the European Economic Area (EEA).
(PDF)

S8 Table *BfArM’s ADR-database* analysis: Reported treatment of ACEi-associated angioedema. S8 Table shows the absolute and relative number of the reported angioedema treatments in the *validated ACEi angioedema cases*. (PDF)
