# Supplementary material for: Angioedemas associated with renin-angiotensin system blocking drugs: Comparative analysis of spontaneous adverse drug reaction reports
Source: PLoS One. 2020 Mar 26;15(3):e0230632. doi: 10.1371/journal.pone.0230632 (PMC7098604; doi:10.1371/journal.pone.0230632)
Supplement: S1 File — (DOCX) [file pone.0230632.s001.docx]

**S1 Document. *EVDAS* analysis: sacubitril/valsartan-associated angioedemas.**

**Introduction**

For sacubitril/valsartan, the angioedema incidence was estimated to be as frequent as for ACEi [1]. The combination product sacubitril/valsartan is an angiotensin receptor and neprilysin inhibitor which acts through the simultaneous inhibition of angiotensin-II-receptors and the inhibition of neprilysin. Neprilysin is also involved in bradykinin degradation [1, 2]. Hence, an increase in bradykinin concentration may be caused by the inhibition of neprilysin through sacubitril. So far, only a few studies have investigated sacubitril/valsartan-associated angioedemas. Therefore, in order to complete the performed analysis of angioedemas associated with drugs acting on the renin-angiotensinsystem, we also analyzed the characteristics, associated factors and clinical phenotypes reported in ADR reports of angioedema associated with the use of sacubitril/valsartan and compared them to their respective controls. In addition, we compared *sacubitril/valsartan angioedema cases* to *ACEi angioedema cases*.

**Methods**

*1) Identification of sacubitril/valsartan angioedema cases*

In **EVDAS** all spontaneous ADR reports registered between 1/2010 -06/2017 within the EEA in which the combination product sacubitril/valsartan was reported as "suspected/interacting" drug were identified (n= 1,429) (query date: 17/12/2018). The angioedema cases were extracted by application of the standardized MedDRA Query (SMQ) "angioedema (narrow)" (n=114) [3]. In order to determine whether there are factors reported more often in *sacubitril/valsartan angioedema cases* we compared them to a group of *controls* consisting of all other sacubitril/valsartan-associated ADR reports excluding *angioedema cases* (n= 1,315).

Analysis of *sacubitril/valsartan angioedema cases* originating from Germany was performed in **EVDAS** (n= 23) since the number of reports varied widely between **BfArM’s ADR-database** and **EVDAS** (more reports in **EVDAS**). This may be explained by a delayed reporting of ADRs after closure of **BfArM’s ADR-database** (22.11.2017). A case validation of all *sacubitril/valsartan angioedema cases* originating from Germany was performed. A more detailed description of the validation process can be found in section 2.2.1) of the manuscript. After case validation, 19 cases (70.4 %) of *sacubitril/valsartan angioedema cases* originating from Germany remained.

*2) Analysis of angioedema cases and controls*

The identified *sacubitril/valsartan angioedema cases* and *controls* were analyzed with regard to reported patient demographics, smoking habits, comorbidities, comedications and the reported seriousness criteria. Comparative analyses were conducted among *sacubitril/valsartan angioedema cases* vs. their *controls* and among *ACEi angioedema cases* vs*. sacubitril/valsartan angioedema cases*. Sacubitril/valsartan was approved in 2016. Hence, the number of sacubitril/valsartan angioedema and ADR (total) reports per 1,000 drug prescriptions in million DDD [4] was calculated based only on the year 2016.

In order to analyze the clinical phenotype, the reports were restricted to those in which only the combination product sacubitril/valsartan was reported as "suspected" which was the case in 92.1 % (n= 105/114) of the European *sacubitril/valsartan angioedema cases*. The diagnosis "angioedema" was only coded in 47.6 % (50/105) of the remaining cases. Hence, to confirm the observed results of the high-level analysis of European *sacubitril/valsartan angioedema cases*, the same analysis was repeated in the full-text analysis of the validated German cases. In the full-text analysis, information about the "time-to-onset" of the angioedema reaction was also retrieved.

**Results**

***1) EVDAS*** *analysis: reported characteristics in sacubitril/valsartan angioedema cases and sacubitril/valsartan controls*

Patients involved in *sacubitril/valsartan angioedema cases* were more often females (OR 1.9 [1.1-3.2]) and younger (OR 0.5 [0.3-0.8]) (patients older than or equal to 65 were coded as 1 and patients younger than 65 coded as 0 in the logistic regression analysis) than patients involved in *sacubitril/valsartan* *controls*. "Allergy" (OR 10.2 [2.8-37.2]), a history of "previous/recurrent angioedema" (3.5 % of cases vs. none of the controls) as well as concurrent ACEi intake (OR 2.6 [1.2-6.0]) were more often reported in *sacubitril/valsartan angioedema cases* vs. *controls*. Although *sacubitril/valsartan angioedema cases* were designated as "serious" slightly more often than their *controls* (OR 1.3 [0.5-3.3]), fatal outcome ("death") was much more frequently reported in *sacubitril/valsartan controls* (OR unadjusted 0.2 [0.1-0.6]). The same applies for "life-threatening" (OR unadjusted 0.7 [0.2-1.9]).

**Table 1) EVDAS** analysis: reported characteristics in *sacubitril/valsartan angioedema cases* and *sacubitril/valsartan controls*

|  | *sacubitril/valsartan angioedema cases* (n= 114; 7.9 %) | *sacubitril/valsartan controls* (n= 1,315; 92.1 %) | unadjusted OR [+/- 95 % CI] | logistic regression OR [+/- 95 % CI] |
| --- | --- | --- | --- | --- |
| *patient demographics*  mean age (median) [years] ^a^  female  male  unknown | 66.2 (66.5)  30.7 % (35)  66.7 % (76)  2.6 % (3) | 70.3 (71.0)  21.7 % (285)  71.4 % (939)  6.9 % (91) | 1.5 [1.0-2.3] | 0.5 [0.3-0.8]*  1.9 [1.1-3.2]* |
| *life style factors, allergic conditions*  smoker ^b^  allergy ^c^ | 3.5 % (4)  4.4 % (5) | 3.0 % (40)  0.8 % (10) | 1.2 [0.4-3.3]  6.0 [2.0-17.8]* | 1.0 [0.3-3.4]  10.2 [2.8-37.2]* |
| *history of skin and subcutaneous disorders ^d^*  urticaria  angioedema | 4.4 % (5)  -  3.5 % (4) | 1.1 % (15)  -  - | 4.0 [1.4-11.1]*  -  - | 5.5 [1.5-19.8]*  -  - |
| *comorbidities ^e^*  renal disease  diabetes  asthma  malignant tumors  thyroid disorders | 4.4 % (5)  9.6 % (11)  1.8 % (2)  2.6 % (3)  0.9 % (1) | 14.3 % (188)  14.8 % (194)  0.6 % (8)  3.0 % (39)  2.4 % (32) | 0.3 [0.1-0.7]*  0.6 [0.3-1.2]  2.9 [0.6-13.9]  0.9 [0.3-2.9]  0.4 [0.0-2.6] | 0.2 [0.1-0.7]*  -  1.2 [0.1-12.3]  0.9 [0.2-4.3]  - |
| *comedication ^f^*  β-blockers  diuretics  calcium antagonists  ACEi  acetylsalicyclic acid  analgesics ^g^  antidiabetics ^h^  DPPIVi  mTORi  fibrinolytics | 26.3 % (30)  34.2 % (39)  3.5 % (4)  8.8 % (10)  13.2 % (15)  2.6 % (3)  6.1 % (7)  0.9 % (1)  -  - | 29.6 % (389)  39.8 % 523)  1.7 % (23)  4.4 % (58)  12.1 % (159)  3.7 % (48)  7.8 % (102)  1.7 % (22)  -  - | 0.9 [0.6-1.3]  0.8 [0.5-1.2]  2.0 [0.7-6.0]  2.1 [1.0-4.2]  1.1 [0.6-1.9]  0.7 [0.2-2.3]  0.8 [0.4-1.7]  0.5 [0.1-3.9]  -  - | 1.1 [0.5-2.5]  0.6 [0.3-1.2]  3.0 [0.8-11.8]  2.6 [1.2-6.0]*  0.9 [0.4-1.9]  0.2 [0.0-1.8]  1.0 [0.4-2.7]  -  -  - |
| *seriousness criteria ^i^*  serious  death  life-threatening  hospitalization  disabling | 92.1 % (105)  4.4 % (5)  3.5 % (4)  26.3 % (30)  - | 89.4 % (1,175)  15.6 % (205)  5.0 % (66)  41.2 % (542)  1.4 % (18) | 1.4 [0.7-2.8]  0.2 [0.1-0.6]*  0.7 [0.2-1.9]  0.5 [0.3-0.8]*  - | 1.3 [0.5-3.3]  -  -  -  - |

*OR=1 is not included; OR > 1 reported more often in *sacubitril/valsartan angioedema cases*; OR < 1 reported more often in *sacubitril/valsartan controls*

^a^ age unknown: *sacubitril/valsartan angioedema cases*: 41 cases (36.0 % of cases), *sacubitril/valsartan controls*: 527 cases (40.1 % of cases).

^b^ refers to current smoking at the time of the reported ADR. Former smokers were classified as non-smokers.

^c^ the term "allergy" refers to a reported allergy and the occurrence of any allergic and hypersensitivity reactions reported in the history of the patient.

^d^ skin and subcutaneous tissue disorders were analyzed based on the SOC "skin and subcutaneous tissue disorders", urticaria based on the HLT "urticarias". The term "angioedema" summarizes previous angioedema or swellings coded in the SMQ "angioedema (narrow)" reported in the history of the patient.

^e^ suitable hierarchical levels of the MedDRA terminology were chosen for analysis of the reported patients’ comorbidities. The term "renal disorders" was identified using the SMQ’s "acute renal failure" and "chronic kidney disease"; "diabetes": SMQ "hyperglycaemia/new onset diabetes mellitus"; "asthma": SMQ "asthma/bronchospasm"; "malignant tumors": SMQ "malignant tumours"; "thyroid disorders": SMQ "thyroid dysfunction".

^f^ the analysis of the most frequently reported and most relevant comedications is based on monosubstances and combination products of the tabulated drug substances and/or drug classes and corresponds to the ATC classification. All drugs co-reported to the "suspected/interacting" ACEi were counted as concomitant, regardless of whether they were reported as "suspected", "interacting" or "concomitant".

^g^ deviating from the ATC-code, the analysis concerning "analgesics" also includes ADR reports in which ibuprofen and/or diclofenac were listed suspected/interacting or concomitant drug. We excluded ADR reports in which acetylsalicyclic acid was listed as suspected/interacting or concomitant drug. The number of ADR reports in which acetylsalicyclic acid was used concurrently were analyzed separately.

^h^ deviating from the ATC-code, we excluded ADR reports in which a DPPIVi was listed as suspected/interacting or concomitant drug in the analysis concerning "diabetics". The number of ADR reports in which DPPIVi was used concurrently was analyzed separately.

^i^ one ADR report may yield information about more than one seriousness criterion, therefore, the number of reported seriousness criteria exceeds that of the ADR reports.

Table 1) shows the absolute and relative number of reports and the calculated unadjusted and adjusted odds ratios for the reported demographic parameters, comorbidities, comedications and seriousness criteria of *sacubitril/valsartan angioedema cases* and *controls* of the European Economic Area (EEA).

*2)* ***EVDAS*** *analysis: characteristics of validated sacubitril/valsartan angioedema cases*

Concerning the calculated completeness score, the *sacubitril/valsartan angioedema cases* were "poorly documented" (Score: 0.50 [0.28-0.73]) compared to *ACEi*, *ARBs* and *aliskiren angioedema cases*. Unfortunately, the number of reports regarding the investigated variables of interest were too small to make any valid statements.

Table 2) **EVDAS** analysis: characteristics of *validated ARBs, aliskiren and sacubitril/valsartan angioedema cases*

|  | *validated sacubitril/valsartan angioedema cases* (n= 19) |
| --- | --- |
| *completeness score* | 0.50 [0.28-0.73] |
| *patient demographics*  mean age (median)  female  male  unknown | 65.4 (72.5)  6 (31.6 %)  13 (68.4 %)  0 (0.0 %) |
| *smoking habits and comorbidities*  smoking ^b^  allergy ^c^  angioedema ^d^  renal disorders ^e^  diabetes ^e^  asthma ^e^ | -  -  1 (5.3 %)  2 (10.5 %)  -  - |
| *comedication ^f^*  β-blockers  diuretics  calcium antagonists  NSAID  everolimus  alteplase  ACEi  ARBs | 5 (26.3 %)  5 (26.3 %)  1 (5.3 %)  3 (15.8 %)  -  -  -  - |
| *seriousness criteria ^g^*  serious  death  life-threatening hospitalization | 19 (100.0 %)  2 (10.5 %)  -  2 (10.5 %) |

^a^ age unknown: *validated sacubitril/valsartan angioedema cases*: 9 cases (47.4 % of cases).

^b^ refers to current smoking at the time of the reported ADR. Former smokers were classified as non-smokers.

^c^ the term "allergy" refers to a reported allergy and the occurrence of any allergic and hypersensitivity reactions reported in the history of the patient.

^d^ the term "angioedema" summarizes previous angioedema or swellings coded in the SMQ "angioedema (narrow)" reported in the history of the patient.

^e^ refers to the respective comorbidity reported in the patients’ history or as a drug indication term for the used comedication.

^f^ the analysis of the most frequently reported and most relevant comedications is based on monosubstances and combination products of the tabulated drug substances and/or drug classes and corresponds to the ATC classification. All drugs co-reported to the respective "suspected/interacting" drug substance were counted as concomitant, regardless of whether they were reported as "suspected", "interacting" or "concomitant".

^g^ one ADR report may yield information about more than one seriousness criterion, therefore, the number of reported seriousness criteria exceeds that of the ADR reports.

Table 2) shows the absolute and relative number of the reported characteristics of *validated sacubitril/valsartan angioedema cases*.

*3)* ***EVDAS*** *analysis: comparative analysis of ACEi angioedema cases* vs. *sacubitril/valsartan angioedema cases.*

More females (OR 1.7 [1.0-2.9]) were included in *sacubitril/valsartan angioedema cases* compared to *ACEi angioedema cases*. Intake of calcium antagonists (OR 9.3 [2.3-38.5]) and analgesics (OR 11.1 [1.5-80.8]) was more frequently reported in *sacubitril/valsartan angioedema cases* vs. *ACEi angioedema cases*. *ACEi angioedema cases* were about 10 times (OR 9.9 [2.4-40.2]) more often designated as "life-threatening" and led to "hospitalization" more than 3 times (OR 3.3 [2.1-5.2]) more often than *sacubitril/valsartan angioedema cases*.

Table 3) **EVDAS** analysis: comparative analysis of *ACEi angioedema cases* vs. *sacubitril/valsartan angioedema cases*.

|  | OR (unadjusted) [+/- 95 % CI] *ACEi angioedema cases* vs. *sacubitril/valsartan angioedema cases* (4 cases excluded ^a^) | logistic regression *ACEi angioedema cases* vs. *sacubitril/valsartan angioedema cases* (4 cases excluded ^a^) |
| --- | --- | --- |
| *patient demographics*  age > 65 years  female | -  1.9 [1.3-2.9]* | 1.1 [0.7-1.9]  1.7 [1.0-2.9]* |
| *smoking habits, allergic conditions and history of skin disorders*  smoker ^b^  allergy ^c^  urticaria ^d^  angioedema ^e^ | 1.1 [0.3-4.6]  0.9 [0.4-2.3]  -  1.5 [0.5-4.8] | 1.1 [0.2-5.1]  0.4 [0.2-1.1]  -  1.1 [0.3-3.6] |
| *comorbidities ^f^*  renal disorders  diabetes  asthma  malignant tumors  thyroid disorders | 1.0 [0.4-2.5]  1.0 [0.5-1.9]  1.3 [0.3-5.3]  1.5 [0.5-4.7]  2.9 [0.4-20.9] | 0.9 [0.3-2.6]  -  -  1.3 [0.3-5.6]  - |
| *comedication ^g^*  β-blockers  diuretics  calcium antagonists  acetylsalicyclic acid  analgesics ^h^  antidiabetics ^i^  DPPIVi  mTORi  fibrinolytics | 0.9 [0.6-1.3]  0.6 [0.4-0.8]  7.5 [2.4-23.9]*  1.7 [1.0-3.0]  4.6 [1.5-14.6]*  1.8 [0.9-3.7]  2.3 [0.3-17.0]  -  - | 0.6 [0.3-1.1]  0.3 [0.2-0.5]*  9.3 [2.3-38.5]*  1.7 [0.9-3.4]  11.1 [1.5-80.8]*  1.3 [0.5-3.1]  -  -  - |
| *seriousness criteria ^j^*  serious  death  life-threatening  hospitalization  disabling | 0.7 [0.4-1.4]  0.3 [0.1-0.9]*  9.9 [2.4-40.2]*  3.3 [2.1-5.2]*  - | 0.8 [0.3-1.9]  -  -  -  - |

*OR=1 is not included; OR > 1 reported more often in *sacubitril/valsartan angioedema cases*; OR < 1 reported more often in *sacubitril/valsartan controls*

^a^ cases which were included in both of the opposing angioedema groups were excluded.

^b^ refers to current smoking at the time of the reported ADR. Former smokers were classified as non-smokers.

^c^ the term "allergy" refers to a reported allergy and the occurrence of any allergic and hypersensitivity reactions reported in the history of the patient.

^d^ the term "urticaria" was analyzed based on the HLT "urticarias".

^e^ the term "angioedema" summarizes previous angioedema or swellings coded in the SMQ "angioedema (narrow)" reported in the history of the patient.

^f^ suitable hierarchical levels of the MedDRA terminology were chosen for the analysis of the reported patients’ comorbidities. The term "renal disorders" was identified using the SMQ’s "acute renal failure" and "chronic kidney disease"; "diabetes": SMQ "hyperglycaemia/new onset diabetes mellitus"; "asthma": SMQ "asthma/bronchospasm"; "malignant tumors": SMQ "malignant tumours"; "thyroid disorders": SMQ "thyroid dysfunction".

^g^ the analysis of the most frequently reported and most relevant comedications is based on monosubstances and combination products of the tabulated drug substances and/or drug classes and corresponds to the ATC classification. All drugs co-reported to the "suspected/interacting" RASi were counted as concomitant, regardless of whether they were reported as "suspected", "interacting" or "concomitant".

^h^ deviating from the ATC-code, the analysis concerning "analgesics" also includes ADR reports in which ibuprofen and/or diclofenac were listed suspected/interacting or concomitant drug. We excluded ADR reports in which acetylsalicyclic acid was listed as suspected/interacting or concomitant drug. The number of ADR reports in which acetylsalicyclic acid was used concurrently were analyzed separately.

^i^ deviating from the ATC-code, we excluded ADR reports in which a DPPIVi was listed as suspected/interacting or concomitant drug in the analysis concerning "diabetics". The number of ADR reports in which DPPIVi was used concurrently was analyzed separately.

^j^ one ADR report may yield information about more than one seriousness criterion, therefore, the number of reported seriousness criteria exceeds that of the ADR reports.

Table 3) shows the calculated unadjusted and adjusted odds ratios of the comparative analysis of *ACEi angioedema cases* vs. *sacubitril/valsartan angioedema cases*.

*4)* ***EVDAS*** *analysis: Number of sacubitril/valsartan angioedema cases in relation to the number of drug prescriptions in Germany (2016)*

The number of *sacubitril/valsartan angioedema cases* in relation to the number of drug prescriptions was higher than for *ACEi, ARBs* and *aliskiren angioedema cases*.

Table 4) Number of *sacubitril/valsartan angioedema cases* and their total number of ADR reports in relation to the number of drug prescriptions in Germany (2016)

| RASi | number of angioedema  reports^a^ (% of all ADR reports for sacubitril/valsartan) | number of all ADR reports  ^a^ | number of drug prescriptions  in Mio DDD ^b^ | number of angioedema reports/number of drug prescriptions in 1,000 Mio DDD (2016) |
| --- | --- | --- | --- | --- |
| *sacubitril/val-sartan* | 23 (8.0 %) | 289 | 3,7 | 6,216 |

^a^ all identified cases (not validated) originating from Germany in **EVDAS** of the year 2016.

^b^ number of drug prescriptions for the combination product sacubitril/valsartan for the year 2016 [4].

Table 4) shows the absolute and relative number of *sacubitril/valsartan angioedema cases* and their total number of ADR reports as well as their relation to the number of drug prescriptions in 1,000 Mio DDD.

*5)* ***EVDAS*** *analysis: reported anatomical area affected by the angioedema*

Regarding the clinical phenotypes the "eye/eyelid" (OR 0.2 [0.1-0.4]) was more often affected in sacubitril/valsartan-associated angioedemas compared to ACEi-associated angioedemas*.* "Urticaria" (9.5 % (n= 10)) as well as "pruritus" (15.2 % (n= 16)) and additional "peripheral swellings/oedemas" (10.5 % (n= 11)) as attendant symptoms were more often reported in *sacubitril/valsartan angioedema cases* compared to *ACEi angioedema cases* (5.0 % (n= 123), 3.1 % (n= 76), 1.2 % (n= 29)).

Table 5) **EVDAS** analysis: reported anatomical area affected by the angioedema and angioedema types according to SMQ "angioedema (narrow)" of the MedDRA terminology.

| rank | *sacubitril/valsartan angioedema cases* (n=105) |
| --- | --- |
| 1. | angioedema (multiple reactions 75.2 %, n=79)  (only 47.6 %, n=50,  OR: 1.3 [0.7-2.5]) |
| 2. | face (17.1 %, n=18)  OR: 0.4 [0.2-1.0] |
| 3. | lips (12.4 %, n=13)  OR: 0.9 [0.3-2.3]  eye/eyelid (12.4 %, n=13)  OR: 0.2 [0.1-0.4]* |
| 4. | urticaria (9.5 %, n=10)  OR: 0.5 [0.2-1.5] |
| 5. | tongue (8.6 %, n=9)  OR: 2.6 [0.8-7.9] |
| 6. | larynx (6.7 %, n=)  OR: 0.4 [0.1-1.4] |
| 7. | mouth (4.8 %, n=5)  OR: 0.4 [0.1-1.7] |
| 8. | pharynx (2.9 %, n=3)  OR: 1.5 [0.2-9.7] |

*OR does not include 1. OR > 1 more often reported in *ACEi angioedema cases*; OR < 1 more often reported in *sacubitril/valsartan angioedema cases.*

Table 5) shows the absolute and relative number of ADR reports for the reported anatomical area affected by the angioedema and angioedema types according to the SMQ "angioedema (narrow)" of the MedDRA terminology, in which the combination product sacubitril/valsartan was reported as "suspected", only. For each anatomical area affected by the angioedema or angioedema type, odds ratios with Bonferroni adjusted confidence intervals were calculated. The *ACEi angioedema cases* served as a reference for the calculation of odds ratios. The number of ADR reports that described the same anatomical area e.g. "tongue oedema" and "swollen tongue" were merged. In some reports only the diagnosis "angioedema" was coded, whereas in some others the term "angioedema" was coded additionally (defined as "angioedema multiple reactions").

6) **EVDAS** analysis: analysis of the "time-to-onset" of sacubitril/valsartan-associated angioedemas

Only nine of 19 *validated sacubitril/valsartan cases* included information about the "time-to-onset" of the angioedema reaction. In 66.7 % (6/9) of these cases the angioedema occurred within the first 30 days of therapy. In the remaining three cases (33.3 % (3/9)) the reaction occurred within the first year of the therapy.

Figure 1**) EVDAS** analysis: "time-to-onset" analysis of the angioedema reaction.

Figure 1 shows the "time-to-onset" analysis of validated sacubitril/valsartan-associated angioedemas compared to validated ACEi, ARBs and aliskiren-associated angioedemas. Only the cases with information provided on the "time-to-onset" were included.

**Discussion**

It seems that some of the reported characteristics like "allergy" and "previous/recurrent angioedema" have a tendency to occur more frequently in *sacubitril/valsartan angioedema cases* compared to their *controls*. However, the case numbers are too small to make a conclusive statement. The same applies to the clinical phenotype and the "time-to-onset" of the angioedema reaction.

According to the calculated completeness score, it seems that the *sacubitril/valsartan angioedema cases* are generally not well documented and are even more poorly documented than *ACEi*, *ARBs* and *aliskiren angioedema cases*. This could also explain the small number of cases regarding the investigated variables of interest. Possibly, the variables of interest were not reported at all.

A higher number of angioedema reports per 1,000 drug prescriptions in million DDD was calculated for sacubitril/valsartan compared to ACEi, ARBs and aliskiren. Sacubitril/valsartan was approved in 2016. Hence, a reporting bias has to be considered. As already mentioned in the discussion of the manuscript, a previous German study investigating ADR reporting behaviors concluded that ADRs related to novel drug therapies are likely to be reported more often than ADRs related to well-known drug therapies [5].

**Conclusion**

The data regarding sacubitril/valsartan-associated angioedemas were insufficient to make any valid statements. Further research with a larger number of cases and/or better documented cases or with other complementary methodological approaches is needed.

**References**

[1] Shi V, Senni M, Streefkerk H, Modgill V, Zhou W, Kaplan A. Angioedema in heart failure patients treated with sacubitril/valsartan (LCZ696) or enalapril in the PARADIGM-HF study. Int J Cardiol. 2018; 264: 118-23.

[2] Hoover T, Lippmann M, Grouzmann E, Marceau F, Herscu P. Angiotensin converting enzyme inhibitor induced angio-oedema: a review of the pathophysiology and risk factors. Clin Exp Allergy. 2010; 40(1): 50-61.

[3] MedDRA: Medical Dictionary for Regulatory Activities. Introductory Guide for Stanardised MedDRA Queries (SMQs) Version 16.0. https://www.meddra.org/sites/default/files/guidance/file/smq_intguide_16_0_english.pdf. Accessed March 2013.

[4] Schwabe U, Paffrath D, Ludwig WD, Klauber J. Arzneiverordnungsreport 2017. Berlin Heidelberg: Springer Verlag Berlin-Heidelberg; 2017.

[5] Hasford J, Goettler M, Munter KH, Müller-Oerlinghausen B. Physicians‘ knowledge and atiitudes regarding the spontaenous reporting system for adverse drug reactions. Journal of Clinical Epidemiology. 2002; 55: 945-950.
