# Supplementary material for: Angioedemas associated with renin-angiotensin system blocking drugs: Comparative analysis of spontaneous adverse drug reaction reports
Source: PLoS One. 2020 Mar 26;15(3):e0230632. doi: 10.1371/journal.pone.0230632 (PMC7098604; doi:10.1371/journal.pone.0230632)
Supplement: S1 Table — * OR = 1 is not included; OR > 1 reported more often in females; OR < 1 reported more often in males. a age unknown: ACEi angioedema cases: 179 cases (5.4% of cases), ACEi controls: 717 cases (6.7% of cases). b refers to current smoking at the time of the reported ADR. Former smokers were classified as non-smokers. c the term "allergy" refers to a reported allergy and the occurrence of any allergic and hypersensitivity reactions reported in the history of the patient. d skin and subcutaneous tissue disorders were analyzed based on the SOC "skin and subcutaneous tissue disorders", urticaria based on the HLT "urticarias". The term "angioedema" summarizes previous angioedema or swellings coded in the SMQ "angioedema (narrow)" reported in the history of the patient. e suitable hierarchical levels of the MedDRA terminology were chosen for analysis of the reported patients’ comorbidities. The term "renal disorders" was identified using the SMQs "acute renal failure" and "chronic kidney disease"; "diabetes": SMQ "hyperglycaemia/new onset diabetes mellitus"; "asthma": SMQ "asthma/bronchospasm"; "malignant tumors": SMQ "malignant tumours"; "thyroid disorders": SMQ "thyroid dysfunction". f the four ACEi monosubstances most frequently reported as "suspected/interacting" are tabulated. The relative number of ADR reports specifying one of the other ACEi (not listed) as "suspected/interacting" was lower than 2%. One ADR report may contain more than one ACEi as "suspected/interacting" drug substance. Thus, the number of reported ACEi exceeds that of the ADR reports. g the analysis of the most frequently reported and most relevant comedications is based on monosubstances and combination products of the tabulated drug substances and/or drug classes and corresponds to the ATC classification. All drugs co-reported to the "suspected/interacting" ACEi were counted as concomitant, regardless of whether they were reported as "suspected", "interacting" or "concomitant". h deviating from th [file pone.0230632.s002.pdf]

S2 Table.

female vs. male	***ACEi angioedema cases: females* (n= 1,506; 47.2 %)**	***ACEi angioedema cases: males* (n= 1,617; 50.6 %)**	**unadjusted OR [+/- 95 % CI]**	**logistic regression OR [+/- 95 % CI]**
patient demographics				
mean age (median) [yeas] [a]	67.1 (69)	66.6 (68)	-	0.9 [0.8-1.1]
smoking habits, allergic conditions				
smoker [b]	1.1 % (17)	3.0 % (49)	0.4 [0.2-0.6]	0.3 [0.2-0.6]*
allergy [c]	6.0 % (89)	2.8 % (46)	2.1 [1.5-3.1]*	2.3 [1.6-3.4]*
history of skin and subcutaneous disorders				
urticaria	0.8 % (12)	0.3 % (5)	2.6 [0.9-7.4]*	3.0 [1.0-9.2]*
angioedema [d]	2.8 % (42)	5.3 % (86)	0.5 [0.4-0.7]*	0.5 [0.3-0.7]*
comorbidities [e]				
renal disorders	3.3 % (50)	5.7 % (92)	0.6 [0.4-0.8]*	0.5 [0.4-0.8]*
diabetes	9.4 % (142)	11.3 % (182)	0.8 [0.7-1.0]	-
asthma	3.3 % (49)	1.5 % (25)	2.1 [1.3-3.5]*	1.8 [1.1-3.1]*
malignant tumors	3.9 % (58)	4.3 % (69)	0.9 [0.8-1.3]	0.9 [0.6-1.3]
thyroid disorders	4.4 % (66)	0.9 % (16)	4.6 [2.6-8.0]	5.6 [3.1-10.0]*
administered ACEi [f]				
ramipril	35.7 % (538)	38.7 % (626)	0.9 [0.8-1.0]	1.0 [0.7-1.4]
enalapril	28.3 % (426)	28.6 % (463)	1.0 [0.8-1.1]	1.0 [0.7-1.4]
perindopril	16.3 % (245)	15.8 % (256)	1.0 [0.9-1.2]	1.1 [0.9-1.3]
lisinopril	14.7 % (221)	11.5 % (186)	1.3 [1.1-1.6]*	1.4 [0.9-2.0]
comedication [g]				
β-blockers	22.1 % (333)	23.8 % (384)	0.9 [0.8-1.1]	1.0 [0.8-1.2]
diuretics	24.4 % (368)	19.8 % (320)	1.3 [1.1-1.6]*	1.5 [1.2-1.8]*
calcium antagonists	15.7 % (236)	19.3 % (312)	0.8 [0.6-0.9]*	0.8 [0.6-0.9]*
ARBs	3.9 % (58)	4.1 % (67)	0.9 [0.6-1.3]	0.9 [0.6-1.3]
acetylsalicyclic acid	16.7 % (251)	23.2 % (375)	0.7 [0.6-0.8]*	0.6 [0.5-0.8]*
analgesics [h]	13.2 % (198)	9.8 % (159)	1.4 [1.1-1.7]*	1.3 [1.1-1.7]*
antidiabetics [i]	10.2 % (153)	10.2 % (165)	1.0 [0.8-1.3]	1.0 [0.8-1.3]
DPPIVi	1.9 % (29)	2.3 % (37)	0.8 [0.5-1.4]	0.9 [0.5-1.5]
mTORi	1.0 % (15)	1.7 % (27)	0.6 [0.3-1.1]	0.8 [0.4-1.5]
fibrinolytics	1.3 % (20)	1.1 % (18)	1.2 [0.6-2.3]	1.1 [0.5-2.2]
seriousness criteria [j]				
serious	87.1 % (1312)	90.0 % (1,456)	0.7 [0.6-0.9]*	0.7 [0.6-0.9]*
death	1.1 % (16)	2.0 % (33)	0.5 [0.3-0.9]*	-
life-threatening	15.1 % (228)	15.9 % (257)	0.9 [0.8-1.1]	-
hospitalization	50.3 % (758)	51.7 % (836)	0.9 [0.8-1.1]	-
disabling	1.0 % (15)	0.7 % (12)	1.3 [0.6-2.9]	-
