# Supplementary material for: Angioedemas associated with renin-angiotensin system blocking drugs: Comparative analysis of spontaneous adverse drug reaction reports
Source: PLoS One. 2020 Mar 26;15(3):e0230632. doi: 10.1371/journal.pone.0230632 (PMC7098604; doi:10.1371/journal.pone.0230632)
Supplement: S2 Table — *OR = 1 is not included; OR > 1 reported more often in ARBs or alsikiren angioedema cases; OR < 1 reported more often in ARBs or aliskiren controls. a age unknown: ARBs angioedema cases: 88 cases (12.8% of cases), ARBs controls: 1,276 cases (14.9% of cases), aliskiren angioedema cases: 37 cases (22.8% of cases), aliskiren controls: 242 cases (23.8% of cases). b only current smoking at the time of the reported ADR was counted. Former smokers were classified as non-smokers. C the term "allergy" summarizes allergic and hypersensitivity reactions reported in the history of the patient. d skin and subcutaneous tissue disorders were analyzed based on the SOC "skin and subcutaneous tissue disorders", urticaria based on the HLT "urticarias". The term "angioedema" summarizes previous angioedema or swellings coded in the SMQ "angioedema (narrow)" reported in the history of the patient. e suitable hierarchical levels of the MedDRA terminology were chosen for the analysis of the reported patients’ comorbidities. The term "renal disorders" was identified using the SMQs "acute renal failure" and "chronic kidney disease"; "diabetes": SMQ "hyperglycaemia/new onset diabetes mellitus"; "asthma": SMQ "asthma/bronchospasm"; "malignant tumors": SMQ "malignant tumours"; "thyroid disorders": SMQ "thyroid dysfunction". f the three ARB monosubstances most frequently reported as "suspected/interacting" are tabulated. One ADR report may contain more than one ARB as "suspected/interacting" drug substance. Thus, the number of reported ARBs exceeds that of the ADR reports. g the analysis of the most frequently reported and most relevant comedications is based on monosubstances and combination products of the tabulated drug substances and/or drug classes and corresponds to the ATC classification. All drugs co-reported to the "suspected/interacting" ARBs were counted as concomitant, regardless of whether they were reported as "suspected", "interacting" or "concomitant". h deviating from the ATC-co [file pone.0230632.s003.pdf]

S3 Table.

***EVDAS* analysis**	**characteristics of *ARBs angioedema cases* and *ARBs controls***		***ARBs angioedema cases* versus *ARBs controls***		**characteristics of *aliskiren angioedema cases* and *aliskiren controls***		***aliskiren angioedema cases* versus *aliskiren controls***	
	ARBs angioedema cases (n= 687; 7.4 %)	*ARBs controls* (n= 8,543 92.6 %)	unadjusted OR [+/- 95 % CI]	logistic regression OR [+/- 95 % CI]	*aliskiren angioedema cases* (n= 162; 13.8 %)	*aliskiren controls* (n= 1,015; 86.2 %)	unadjusted OR [+/- 95 % CI]	logistic regression OR [+/- 95 % CI]
patient demographics								
mean age (median) [years] [a]	65.5 (67.0)	67.3 (69.0)	-	0.9 [0.7-1.0]	64.7 (68.0)	67.6 (70.0)	-	1.0 [0.6-1.5]
female	57.1 % (392)	54.0 % (4,615)	1.1 [1.0-1.3]	1.1 [0.9-1.3]	61.1 % (99)	47.7 % (484)	1.8 [1.3-2.5]*	1.5 [1.0-2.2]
male	40.2 % (276)	42.5 % (3,633)			35.8 % (58)	49.8 % (505)		
unknown	2.8 % (19)	3.5 % (295)			3.1 % (5)	2.6 % (26)		
smoking habits, allergic conditions								
smoker [b]	2.0 % (14)	1.7 % (143)	1.2 [0.7-2.1]	1.3 [0.7-2.3]	1.2 % (2)	2.5 % (25)	0.5 [0.1-2.1]	0.6 [0.1-2.8]
allergy [c]	6.8 % (47)	2.6 % (224)	2.7 [2.0-3.8]*	1.5 [1.0-2.3]*	13.6 % (22)	3.3% (33)	4.7 [2.7-8.3]*	3.5 [1.7-7.2]*
history of skin and subcutaneous tissue disorders [d]	7.0 % (48)	2.0 % (173)	3.6 [2.6-5.1]*	-	6.2 % (10)	2.3 % (23)	2.8 [1.3-6.1]*	1.3 [0.5-3.4]
urticaria	0.9 % (6)	0.1 % (8)	9.4 [3.3-27.2]*	4.8 [1.5-15.7]*	-	-	-	-
angioedema	4.5 % (31)	0.1 % (11)	36.7 [18.3-73.3]*	22.7 [10.6-48.3]*	4.9 % (8)	0.1 % (1)	52.7 [6.5-424.1]*	-
comorbidities [e]								
renal disease	2.0 % (14)	5.7 % (492)	0.3 [0.2-0.6]*	0.4 [0.2-0.6]*	4.9 % (6)	15.3 % (116)	0.3 [0.1-0.7]*	0.4 [0.2-1.0]
diabetes	7.6 % (52)	9.4 % (807)	0.8 [0.6-1.1]	-	12.3 % (20)	18.6 % (189)	0.6 [0.4-1.0]	-
asthma	2.9 % (20)	1.6 % (133)	1.9 [1.2-3.1]*	1.3 [0.8-2.4]	4.9 % (8)	1.3 % (13)	4.0 [1.6-9.8]*	5.0 [1.7-14.8]*
malignant tumors	2.9 % (20)	4.5 % 386)	0.6 [0.4-1.0]	0.6 [0.4-1.0]	2.5 % (4)	3.6 % (37)	0.7 [0.2-1.9]	0.5 [0.1-1.9]
thyroid disorders	3.3 % (23)	3.0 % (259)	1.1 [0.7-1.7]	1.2 [0.7-2.0]	4.3 % (7)	2.2 % (22)	2.0 [0.9-4.9]	2.8 [1.0-7.9]
administered ARBs [f]								
losartan	25.8 % (177)	19.1 % (1,633)	1.5 [1.2-1.8]*	1.7 [1.4-2.1]*	-	-	-	-
candesartan	24.9 % (171)	21.0 % (1,792)	1.2 [1.0-1.5]	1.7 [1.3-2.1]*	-	-	-	-
valsartan	22.4 % 154)	21.7 % (1,855)	1.0 [0.9-1.3]	1.4 [1.1-1.8]*	-	-	-	-
comedication [g]								
β-blockers	18.5 % (127)	23.5 % (2,010)	0.7 [0.6-0.9]*	0.8 [0.7-1.0]	18.5 % (30)	30.0 % (304)	0.5 [0.3-0.8]*	0.7 [0.4-1.2]

diuretics	20.4 % (140)	29.0 % (2,481)	0.6 [0.5-0.8]*	0.6 [0.5-0.7]*	23.5 % (38)	37.2 % (378)	0.5 [0.4-0.8]*	0.6 [0.4-1.0]
calcium antagonists	15.4 % 106)	16.9 % (1,443)	0.9 [0.7-1.1]	0.9 [0.7-1.1]	28.4 % (46)	28.2 % (286)	1.0 [0.7-1.5]	1.5 [0.9-2.4]
ACEi	10.2 % (70)	4.6 % (397)	2.3 [1.8-3.0]*	2.2 [1.6-3.0]*	11.1 % (18)	17.8 % (181)	0.6 [0.3-1.0]	0.6 [0.3-1.2]
ARBs	-	-	-	-	11.7 % (19)	22.7 % (230)	0.5 [0.3-0.7]*	0.5 [0.3-1.0]
acetylsalicyclic acid	12.1 % 83)	13.8 % (1,176)	0.9 [0.7-1.1]	1.1 [0.9-1.5]	14.8 % (24)	13.5 % (137)	1.1 [0.7-1.8]	1.8 [1.0-3.2]
analgesics [h]	8.6 % (59)	10.7 % (913)	0.8 [0.6-1.0]	0.7 [0.5-1.0]*	6.2 % (10)	7.1 % (72)	0.9 [0.4-1.7]	0.5 [0.2-1.2]
antidiabetics [i]	7.0 % (48)	9.4 % (803)	0.7 [0.5-1.0]	0.8 [0.5-1.1]	7.4 % (12)	13.6 % (138)	0.5 [0.3-0.9]*	0.6 [0.3-1.2]
DPPIVi	2.9 % (20)	1.7 % (142)	1.8 [1.1-2.9]*	1.8 [1.1-3.1]*	3.7 % (6)	2.0 % (20)	1.9 [0.8-4.8]	1.6 [0.5-5.2]
mTORi	0.3 % (2)	0.2 % (16)	1.6 [0.4-6.8]	2.3 [0.5-10.6]	-	0.1 % (1)	-	-
fibrinolytics	0.1 % (1)	-	-	-	-	-	-	-
seriousness criteria [j]								
serious	81.7 % (561)	71.4 % (6,102)	1.8 [1.5-2.2]*	1.8 [1.5-2.3]*	96.9 % (157)	91.8 % (932)	2.8 [1.1-7.0]*	3.1 [1.2-7.9]*
death	0.6 % (4)	2.5 % (215)	0.2 [0.1-0.6]*	-	2.5 % (4)	4.9 % (50)	0.5 [0.2-1.4]	-
life-threatening	8.6 % (59)	4.9 % (419)	1.8 [1.4-2.4]*	-	1.2 % (2)	6.1 % (62)	0.2 [0.0-0.8]*	-
hospitalization	32.0 % (220)	38.8 % (3,315)	0.7 [0.6-0.9]*	-	16.7 % (27)	37.1 % (377)	0.3 [0.2-0.5]*	-
disabling	2.9 % (20)	2.6 % (218)	1.1 [0.7-1.8]	-	1.2 % (2)	1.9 % (19)	0.7 [0.2-2.8]	-
