# Supplementary material for: Angioedemas associated with renin-angiotensin system blocking drugs: Comparative analysis of spontaneous adverse drug reaction reports
Source: PLoS One. 2020 Mar 26;15(3):e0230632. doi: 10.1371/journal.pone.0230632 (PMC7098604; doi:10.1371/journal.pone.0230632)
Supplement: S3 Table — a the calculation of the mean number of angioedema reports, controls (ADR reports without angioedema reports) and ADR reports (total) per 1 million ACEi-exposed inhabitants/males/females was restricted to the years 2010–2016. This was the case since the ADR reports were analyzed for only half of the year 2017 (analysis criteria 01/01/2010-30/06/2017). S3 Table shows the calculated mean number of angioedema and ADR reports in relation to the assumed number of ACEi-exposed inhabitants/males/females per 1 million assumed ACEI-exposed inhabitants/males/females in Germany. The number of inhabitants per year was extracted from the GENESIS database [50] and multiplied by the proportional share of ACEi exposure in the German population published in DEGS1 [33]. A proportion of about 17.5% of German adults, 19.0% of German adult males, and 16.0% of German adult females taking an ACEi were extracted from the published graphic in DEGS1. (PDF) [file pone.0230632.s004.pdf]

S4 Table.

	**mean number of angioedema reports [a] per 1 million assumed ACEi-exposed inhabitants/ males/ females**	**mean number of controls (ADR reports [a] without angioedema reports) per 1 million assumed ACEi-exposed inhabitants/ males/ females**	**mean number of ADR reports (total) [a] per 1 million assumed ACEi-exposed inhabitants/ males/ females**
total	3 angioedema reports	10 controls	13 ADR reports (total)
male	2 angioedema reports	8 controls	10 ADR reports (total)
female	3 angioedema reports	12 controls	14 ADR reports (total)
