# Supplementary material for: Angioedemas associated with renin-angiotensin system blocking drugs: Comparative analysis of spontaneous adverse drug reaction reports
Source: PLoS One. 2020 Mar 26;15(3):e0230632. doi: 10.1371/journal.pone.0230632 (PMC7098604; doi:10.1371/journal.pone.0230632)
Supplement: S4 Table — *OR = 1 is not included; OR > 1 reported more often in matched validated ACEi angioedema cases; OR < 1 reported more often in matched ACEi controls (not validated) a in 7 of the validated ACEi angioedema cases neither age or gender (or both) were reported, hence 114 cases remained. The 1:2 matching by age and gender to the ACEi controls (not validated) was only performed for the cases in which age and gender were reported. b refers to current smoking at the time of the reported ADR. Former smokers were classified as non-smokers. c the term "allergy" refers to a reported allergy and the occurrence of any allergic and hypersensitivity reactions reported in the history of the patient. d the term "angioedema" summarizes previous angioedema, or swellings coded in the SMQ "angioedema (narrow)" reported in the history of the patient. e refers to the respective comorbidity reported in the patients’ history or as a drug indication tem for the used comedication. f the analysis of the most reported and most relevant comedications is based on monosubstances and combination products of the tabulated drug substances and/or drug classes and corresponds to the ATC classification. All drugs co-reported to the "suspected/interacting" ACEi were counted as concomitant, irrespective if they were reported as "suspected", "interacting", or "concomitant". g one ADR report may inform about more than one seriousness criterion. Thus, the number of reported seriousness criteria exceeds the number of ADR reports. S4 Table shows the absolute and relative number of reports and the calculated unadjusted odds ratios for the reported demographic parameters, comorbidities, comedications, and seriousness criteria of the matched validated ACEi angioedema cases and matched ACEi controls (not validated). (PDF) [file pone.0230632.s005.pdf]

S5 Table.

	matched validated ACEi angioedema cases **(n= 114)**[a]	*matched ACEi controls (not validated)* **(n= 228)**	**unadjusted OR [+/- 95 % CI)**
patient demographics			
mean age (median) [years]	64.5 (68)	64.5 (67.5)	-
female/male	51 (44.7 %) / 63 (55.3 %)	102 (44.7 %) / 126 (55.3 %)	-
smoking habits, allergic conditions			
smoking [b]	17 (14.9 %)	9 (3.9 %)	4.3 [1.8-9.9]*
allergy [c]	14 (12.3 %)	16 (7.0 %)	1.9 [0.9-3.9]
skin and subcutaneous disorders	8 (7.0 %)	7 (3.1 %)	-
urticaria	1 (0.9 %)	0 (0.0 %)	-
angioedema [d]	26 (22.8 %)	0 (0.0 %)	-
comorbidities [e]			
renal disorders	12 (10.5 %)	20 (8.8 %)	1.2 [0.6-2.6]
diabetes	19 (16.7 %)	45 (19.7 %)	0.8 [0.5-1.5]
asthma	5 (4.4 %)	4 (1.8 %)	2.6 [0.7-9.8]
malignant tumors	6 (5.3 %)	13 (5.7 %)	0.9 [0.3-2.5]
thyroid disorders	11 (9.6 %)	15 (6.6 %)	1.5 [0.7-3.4]
comedication [f]			
β-blockers	34 (29.8 %)	71 (31.1 %)	0.9 [0.6-1.5]
diuretics	15 (13.1 %)	59 (25.9 %)	0.4 [0.2-0.8]*
calcium antagonists	19 (16.7 %)	37 (16.2 %)	1.0 [0.6-1.9]
acetylsalicyclic acid	23 (20.2 %)	43 (18.9 %)	1.1 [0.6-1.9]
NSAID	4 (3.5 %)	10 (4.4 %)	0.8 [0.2-2.6]
diabetics	15 (13.1 %)	37 (16.2 %)	0.8 [0.4-1.5]
everolimus	6 (5.3 %)	0 (0.0 %)	-
alteplase	1 (0.9 %)	0 (0.0 %)	-
seriousness criteria [g]			
serious	101 (88.6 %)	161 (70.6 %)	3.2 [1.7-6.2]*
death	4 (3.5 %)	4 (1.8 %)	2.0 [0.5-8.3]
life-threatening	33 (28.9 %)	25 (11.0 %)	3.3 [1.9-5.9]*
hospitalization	58 (50.9 %)	81 (35.5 %)	1.9 [1.2-3.0]*
