# Supplementary material for: Angioedemas associated with renin-angiotensin system blocking drugs: Comparative analysis of spontaneous adverse drug reaction reports
Source: PLoS One. 2020 Mar 26;15(3):e0230632. doi: 10.1371/journal.pone.0230632 (PMC7098604; doi:10.1371/journal.pone.0230632)
Supplement: S5 Table — a age unknown: validated ARBs angioedema cases: 21 cases (33.3% of cases), validated aliskiren angioedema cases: 13 cases (39.4% of cases). b refers to current smoking at the time of the reported ADR. Former smokers were classified as non-smokers. c the term "allergy" refers to a reported allergy and the occurrence of any allergic and hypersensitivity reactions reported in the history of the patient. d the term "angioedema" summarizes previous angioedema, or swellings coded in the SMQ "angioedema (narrow)" reported in the history of the patient. e refers to the respective comorbidity reported in the patients’ history or as a drug indication tem for the used comedication. f the analysis of the most reported and most relevant comedications is based on monosubstances and combination products of the tabulated drug substances and/or drug classes and corresponds to the ATC classification. All drugs co-reported to the respective "suspected/interacting" drug substance were counted as concomitant, irrespective if they were reported as "suspected", "interacting", or "concomitant". g one ADR report may inform about more than one seriousness criterion. Thus, the number of reported seriousness criteria exceeds the number of ADR reports. h one ADR report may inform about more than one anatomical area affected of the angioedema. Thus, the number of reported anatomical areas affected of the angioedema exceeds the number of ADR reports. i one ADR report may inform about more than one attendant symptom. Thus, the number of reported attendant symptoms exceeds the number of ADR reports. S5 Table shows the absolute and relative number of the reported characteristics of the validated ARBs and aliskiren angioedema cases. (PDF) [file pone.0230632.s006.pdf]

S6 Table.

	***validated ARBs angioedema cases* (n= 63)**	***validated aliskiren angioedema cases* (n= 33)**
completeness score	0.67 [0.54-0.80]	0.68 [0.49-0.88]
patient demographics		
mean age (median) [years]	66.2 (69)	62.1 (66)
female	45 (71.4 %)	21 (63.6 %)
male	17 (27.0 %)	11 (33.3 %)
unknown	1 (1.6 %)	1 (3.0 %)
smoking habits and comorbidities		
smoking [b]	2 (3.2 %)	1 (3.0 %)
allergy [c]	12 (19.0 %)	8 (24.2 %)
angioedema [d]	7 (11.1 %)	12 (36.4 %)
renal disorders [e]	-	2 (6.1 %)
diabetes [e]	3 (4.8 %)	7 (21.2 %)
asthma [e]	2 (3.2 %)	1 (3.0 %)
comedication [f]		
β-blockers	11 (17.5 %)	7 (21.2 %)
diuretics	8 (12.7 %)	7 (21.2 %)
calcium antagonists	10 (15.9 %)	7 (21.2 %)
NSAID	3 (4.8 %)	1 (3.0 %)
everolimus	-	-
alteplase	-	-
ACEi	-	5 (15.2 %)
ARBs	-	3 (9.1 %)
seriousness criteria [g]		
serious	57 (90.5 %)	161 (70.6 %)
death	-	4 (1.8 %)
life-threatening	3 (4.8 %)	25 (11.0 %)
hospitalization	15 (23.8 %)	81 (35.5 %)
anatomical area affected by the angioedema [h]		
tongue	13 (20.6 %)	5 (15.2 %)
lips	13 (20.6 %)	8 (24.4 %)
face	22 (34.9 %)	13 (39.4 %)
pharynx	4 (6.3 %)	2 (6.1 %)
larynx	3 (4.8 %)	1 (3.0 %
eye/eyelid	8 (12.7 %)	4 (12.1 %)
throat	8 (12.7 %)	3 (9.1 %)
urticaria	8 (12.7 %)	3 (9.1 %)
reported attendant reactions [i]		
pruritus	10 (15.9 %)	5 (15.2 %)
peripheral swellings/oedemas	1 (1.6 %)	4 (12.1 %)
prior history of ACEi and/or ARBs use	8 (12.7 %)	13 (39.4 %)
withdrawn due to "cough"	4/8 (50.0 %)	2/ 13 (15.4 %)
withdrawn due to "allergy"	1/8 (12.5 %)	-
withdrawn due to "angioedema"	1/8 (12.5 %)	7/13 (53.8 %)
reason for withdrawn "NA"	2/8 (25.0 %)	4/13 (30.8 %)
