# Supplementary material for: Angioedemas associated with renin-angiotensin system blocking drugs: Comparative analysis of spontaneous adverse drug reaction reports
Source: PLoS One. 2020 Mar 26;15(3):e0230632. doi: 10.1371/journal.pone.0230632 (PMC7098604; doi:10.1371/journal.pone.0230632)
Supplement: S6 Table — a all identified cases (not validated) in BfArM’s ADR-database analysis of the time period 01/2010-12/2016. b cumulative number of drug prescriptions (monosubstances) for the years 2010–2016 [34]. c all angioedema reports including reports from 2017. The administered reported dose was analyzed during the validation process based on the complete report (including narratives; see Material and methods). d definition of ATC-code and the respective DDD of ACEi, ARBs and aliskiren monosubstances [41, 42]. e the incidences were taken from a meta-analysis of randomized trials performed by Makani et al. [23]. f number of ACEi reports with concomitant use of everolimus. g number of drug prescriptions for everolimus [34]. S6 Table shows the absolute and relative number of ACEi, ARBs and aliskiren angioedema cases and their total number of ADR reports in the time periode 01/2010-12/2016 as well as their relation to the number of drug prescriptions in 1,000 Mio DDD. Additionally, the number of angioedema reports per drug prescriptions fitted to the administered dose versus defined daily dose (DDD) ratio was calculated. (PDF) [file pone.0230632.s007.pdf]

**S7 Table.**

| *RASi* | **number of angioedema reports [a] (% of all ADR reports of the respective drug substance)** | **number of all ADR reports [a]** | **number of drug prescriptions in Mio DDD [b]** | **number of angioedema reports / number of drug prescriptions in 1,000 Mio DDD** | **mean administered dose (median) [mg] [c]** | **defined daily dose (DDD) [mg] [d]** | **administered dose/ DDD ratio** | **number of angioedema reports/number of drug prescriptions in 1,000 Mio of the administered dose** | **meta-analysis of randomized trials of angioedema as an adverse event of renin-angiotensin system inhibitors [e]** |
|---|---|---|---|---|---|---|---|---|---|
| ***ACEi*** | 253 (20.3 %) | 1246 | 32382,4 | 8 | - | - | - | - | 0.30 % |
| **ramipril** | 181 (20.2 %) | 896 | 25846,8 | 7 | 6.0 (5.0) | 2.5 | 2.4 (2) | 17 | - |
| **lisinopril** | 28 (25.9 %) | 108 | 2199,3 | 13 | 15.8 (15.0) | 10.0 | 1.6 (1.5) | 20 | - |
| **enalapril** | 33 (17.4 %) | 190 | 3741,1 | 9 | 11.7 (5.0) | 10.0 | 1.2 (0.5) | 10 | - |
| **ACEi + everolimus** | 10 (90.9 %) | 11 [f] | 11,9 [g] | 840 | - | - | - | - | - |
| ***ARBs*** | 103 (7.6 %) | 1361 | 10550,7 | 10 | - | - | - | - | 0.11 % |
| **valsartan** | 48 (10.5 %) | 455 | 3089,2 | 16 | 126.6 (160.0) | 80.0 | 1.6 (2.0) | 25 | - |
| **candesartan** | 32 (6.8 %) | 469 | 4720,3 | 7 | 17.1 (16.0) | 8.0 | 2.1 (2.0) | 14 | - |
| **losartan** | 10 (8.0 %) | 125 | 868,6 | 12 | 75.0 (75.0) | 50.0 | 1.5 (1.5) | 17 | - |
| ***aliskiren*** | 50 (12.7 %) | 394 | 267,6 | 154 | 204.0 (150.0) | 150.0 | 1.4 (1.0) | 252 | 0.13 % |
