# Supplementary material for: Angioedemas associated with renin-angiotensin system blocking drugs: Comparative analysis of spontaneous adverse drug reaction reports
Source: PLoS One. 2020 Mar 26;15(3):e0230632. doi: 10.1371/journal.pone.0230632 (PMC7098604; doi:10.1371/journal.pone.0230632)
Supplement: S7 Table — a age unknown: ACEi angioedema cases with concomitant mTORi therapy: 3 cases (7.1% of cases), ACEi angioedema cases with concomitant fibrinolytics therapy: 2 cases (5.3% of cases), ACEi angioedema cases with concomitant DPPIVi therapy: 6 cases (9.0% of cases). b current smoking at the time of the reported ADR was count, only. Former smokers were classified as non-smokers. c the term "allergy" summarizes allergic and hypersensitivity reactions reported in the history of the patient. d skin and subcutaneous tissue disorders were analyzed based on the SOC "skin and subcutaneous tissue disorders", urticaria based on the HLT "urticarias". The term "angioedema" summarizes previous angioedema, or swellings coded in the SMQ "angioedema (narrow)" reported in the history of the patient. e suitable hierarchical levels of the MedDRA terminology were chosen for the analysis of the reported patients’ comorbidities. The term "renal disorders" was identified using the SMQs "acute renal failure" and "chronic kidney disease"; "diabetes": SMQ "hyperglycaemia/new onset diabetes mellitus"; "asthma": SMQ "asthma/bronchospasm"; "malignant tumors": SMQ "malignant tumours"; "thyroid disorders": SMQ "thyroid dysfunction". f tabulated are the four ACEi monosubstances reported as "suspected/interacting" most frequently (of all cases). One ADR report may contain more than one ACEi as "suspected/interacting" drug substance. Thus, the number of reported ACEi exceeds the number of ADR reports. g the analysis of the most frequently reported and most relevant comedications is based on monosubstances and combination products of the tabulated drug substances and/or drug classes and corresponds to the ATC classification. All drugs co-reported in ACEi angioedema cases with concurrent mTORi, fibrinolytics or DPPIVi use were counted as concomitant, regardless of whether they were reported as "suspected", "interacting" or "concomitant". h deviating from the ATC-code, the analysis concerning "analgesics" al [file pone.0230632.s008.pdf]

S8 Table.

	ACEi angioedema cases **with mTORi as concomitant drug (n= 42; 1.3 %)**	***ACEi angioedema cases*** **with fibrinolytics as concomitant drug (n= 38; 1.2 %)**	***ACEi angioedema cases*** **with DPPIVi as concomitant drug (n= 67; 2.1 %)**
patient demographics			
mean age (median) [years] [a]	62.6 (62)	71.6 (74)	68.3 (67)
female	35.7 % (15)	52.6 % (20)	43.3 % (29)
male	64.3 % (27)	47.4 % (18)	55.2 % (37)
unknown	0.0 % (0)	0.0 % (0)	1.5 % (1)
smoking habits, allergic conditions			
smoker [b]	2.3 % (1)	5.3 % (2)	3.0 % (2)
allergy [c]	0.0 % (0)	0.0 % (0)	4.5 % (3)
history of skin and subcutaneous disorders			
urticaria	0.0 % (0)	0.0 % (0)	1.5 % (1)
angioedema [d]	7.1 % (3)	2.6 % (1)	9.0 % (6)
comorbidities [e]			
renal disorders	31.0 % (13)	0.0 % (0)	6.0 % (4)
diabetes	11.9 % (5)	36.8 % (14)	61.2 % (41)
asthma	0.0 % (0)	2.6 % (1)	3.0 % (2)
malignant tumors	28.6 % (12)	2.6 % (1)	9.0 % (6)
thyroid disorders	4.7 % (2)	13.2 % (5)	4.5 % (3)
administered ACEi [f]			
ramipril	57.1 % (24)	18.4 % (7)	50.8 % (34)
enalapril	14.3 % (6)	26.3 % (10)	16.4 % (11)
perindopril	11.9 % (5)	26.3 % (10)	20.9 % (14)
lisinopril	11.9 % (5)	10.5 % (4)	10.5 % (7)
comedication [g]			
β-blockers	31.0 % (13)	34.2 % (13)	29.9 % (20)
diuretics	26.2 % (11)	26.3 % (10)	35.8 % (24)
calcium antagonists	7.1 % (3)	18.4 % (7)	17.9 % (12)
ARBs	0.0 % (0)	2.6 % (1)	3.0 % (2)
acetylsalicyclic acid	23.8 % (10)	18.4 % (7)	37.3 % (25)
analgesics [h]	2.4 % (1)	5.3 % (2)	16.4 % (11)
antidiabetics [i]	9.5 % (4)	31.6 % (12)	0.0 % (0)
DPPIVi	0.0 % (0)	2.6 % (1)	-
mTORi	-	0.0 % (0)	0.0 % (0)
fibrinolytics	0.0 % (0)	-	1.5 % (1)
seriousness criteria [j]			
serious	92.9 % (39)	86.8 % (33)	98.5 % (66)
death	4.8 % (2)	2.6 % (1)	6.0 % (4)
life-threatening	9.5 % (4)	50.0 % (19)	22.4 % (15)
hospitalization	59.5 % (25)	26.3 % (10)	67.2 % (45)
disabling	0.0 % (0)	0.0 % (0)	0.0 % (0)
anatomical area affected by the angioedema [k]			
angioedema	69.0 % (29)	71.1 % (27)	83.6 % (56)
tongue	31.0 % (13)	31.6 % (12)	20.9 % (14)
lips	4.8 % (2)	10.5 % (4)	4.5 % (3)
face	21.4 % (9)	2.6 % (1)	9.0 % (6)

pharynx	9.5 % (4)	5.3 % (2)	1.5 % (1)
larynx	2.4 % (1)	2.6 % (1)	4.5 % (3)
palatal	0.0 % (0)	0.0 % (0)	0.0 % (0)
mouth	4.8 % (2)	0.0 % (0)	1.5 % (1)
eye/eyelid	0.0 % (0)	0.0 % (0)	1.5 % (1)
urticaria	0.0 % (0)	0.0 % (0)	10.4 % (7)
reported attendant reactions [l]			
dyspnoea	11.9 % (5)	15.8 % (6)	10.4 % (7)
pruritus	-	-	1.5 % (1)
hypersensitivity	-	2.6 % (1)	3.0 % (2)
dysphagia	2.4 % (1)	-	1.5 % (1)
rash	-	-	-
erythema	2.4 % (1)	-	1.5 % (1)
