# Supplementary material for: Angioedemas associated with renin-angiotensin system blocking drugs: Comparative analysis of spontaneous adverse drug reaction reports
Source: PLoS One. 2020 Mar 26;15(3):e0230632. doi: 10.1371/journal.pone.0230632 (PMC7098604; doi:10.1371/journal.pone.0230632)
Supplement: S8 Table — (PDF) [file pone.0230632.s009.pdf]

S9 Table.

reported angioedema treatment	***validated ACEi angioedema cases* (n= 78; 64.4 %)**
Antihistamine and/or steroids	60.3 % (47/78)
additional cooling	4.3 % (2/47)
antihistamines only	8.5 % (4/47)
steroids only	38.3 % (18/47)
rapid regression of symptoms	31.9 % (15/47)
slow regression of symptoms	27.7 % (13/47)
regression was not assessable or not available	40.4 % (19/47)
circulation stabilizing drugs	12.8 % (10/78)
additional with antihistamines/steroids	90% (9/10)
circulation stabilizing drugs only	10 % (1/10)
rapid regression of symptoms	20 % (2/10)
slow regression of symptoms	70 % (7/10)
regression was not assessable or not available	10 % (1/10)
medical intervention	16.7 % (13/78)
additional with other medications	53.8 % (7/13)
medical intervention only	46.2 % (6/13)
intubation	61.5% (8/13)
conitotomy	15.4 % (2/13)
tracheotomy	15.4 % (2/13)
laryngeal tube	7.7 % (1/13)
C1-esterase inhibitors	10.3 % (8/78)
additional with antihistamines/steroids	75.0 % (6/8)
C1-esterase inhibitors only	25.0 % (2/8)
rapid regression of symptoms	75 % (6/8)
slow regression of symptoms	12.5 % (1/8)
regression was not assessable or not available	12.5 % (1/8)
icatibant	5.1 % (4/78)
additional with antihistamines/steroids	50.0 % (2/4)
icatibant only	50.0 % (2/4)
rapid regression of symptoms	100.0 % (4/4)
slow regression of symptoms	-
fresh frozen plasma	1.3 % (1/78)
additional with antihistamines/steroids	100.0 % (1/1)
rapid regression of symptoms	100.0 % (1/1)
slow regression of symptoms	-
